# Metagenomic monitoring of soil bacterial community after the construction of a crude oil flowline

**DOI:** 10.1007/s10661-021-09637-3

**Published:** 2022-01-03

**Authors:** Maria Grazia Bonomo, Luana Calabrone, Laura Scrano, Sabino Aurelio Bufo, Katia Di Tomaso, Euro Buongarzone, Giovanni Salzano

**Affiliations:** 1 Department of Sciences, University of Basilicata, Potenza, Italy; 2 Department of European Cultures, University of Basilicata, Potenza, Italy; 3 Department of Geography, Environmental Management and Energy Studies, University of Johannesburg, Johannesburg, South Africa; 4 SAIPEM S.P.A, Fano, Italy

**Keywords:** Soil bacterial diversity, Microbial composition changes, Metagenomic analyses, Oil flowline effects

## Abstract

This study aimed to assess the metagenomic changes of soil bacterial community after constructing a crude oil flowline in Basilicata region, Italy. Soils identified a total of 56 taxa at the phylum level and 485 at the family level, with a different taxa distribution, especially in samples collected on 2014. Since microbiological diversity occurred in the soils collected after 2013 (the reference year), we performed a differential abundance analysis using DESeq2 by GAIA pipeline. In the forest area, 14 phyla and 126 families were differentially abundant (− 6.06 < logFC > 7.88) in 2014 compared to 2013. Nine families were differentially abundant in 2015, with logFC between − 3.16 and 4.66, while 20 families were significantly more abundant and 16 less abundant in 2016, with logFC between − 6.48 and 6.45. In the cultivated area, 33 phyla and 260 families showed differential abundance in 2014. In the next year (2015), 14 phyla were significantly more abundant and 19 less abundant, while 29 families were substantially more abundant and 139 less abundant, with fold changes ranging between − 5.67 and 4.01. In 2016, 33 phyla showed a significantly different abundance, as 14 were more abundant and 19 decreased, and 81 families showed a significantly increased amount with logFC between − 5.31 and 5.38. These results hypothesise that the analysed site is an altered soil where the development of particular bacterial groups attends to bioremediation processes, naturally occurring to restore optimal conditions.

**Supplementary Information:**

The online version contains supplementary material available at 10.1007/s10661-021-09637-3.

## Introduction

Soil is a rich and dynamic ecosystem representing the most vast microbial diversity source on the entire world. This hidden biodiversity could be a great resource of natural products for agriculture and biotechnological applications (Mocali & Benedetti, 2010; Ahmed et al., 2018).

Soil microorganisms play a crucial role in ecologically critical biogeochemical processes, contributing to plant nutrition and soil health, even in agricultural and extreme environments, and maintaining the matter and energy transfer in terrestrial environments (Arias et al., 2005; Lelario et al., 2018; Mader et al., 2002; Sofo et al., 2018). Soil represents, with its composite microbial functions, a very complex and heterogeneous environment for microbiologists. Due to soil properties and interaction processes, involving mineral and organic particles, soil biota gives rise to the formation and stabilisation of differently sized aggregates, micropores and clay-organic matter complexes that dominate the soil characteristics and affect the microbial composition (Mocali & Benedetti, 2010).

Besides, microbial communities in soil are randomly spread out, following the best growing conditions, such as nutrient gradients and moisture content, and leading to the so-called hot-spot distribution (Mocali & Benedetti, 2010). Most soil microbial communities represent a new source of genetic and metabolic diversity; in fact, only a small fraction of the soil bacteria (less than 1%) is cultivable, highlighting the need to assess and preserve the diversity in soil microorganisms’ distribution (Mocali & Benedetti, 2010).

Traditional microbiological approaches present severe limitations to knowledge of soil microbial diversity (Mocali & Benedetti, 2010; Torsvik & Ovreas, 2002). Therefore, in the last decades, molecular fingerprinting techniques were developed, representing a rapid and powerful tool for understanding soil microbial communities’ dynamics and diversity (Bonomo & Salzano, 2013; Bonomo et al., 2013; van Elsas et al., 2007). However, these approaches proved limitations and biases, mainly related to the target gene’s characteristics and PCR amplification efficiency, which have always limited knowledge to a restricted part of the microbial communities (Kirk et al., 2004; Cafaro et al., 2016). For these reasons, novel approaches to exploring the vast majority of soil microbial diversity were necessary (Mocali & Benedetti, 2010).

In recent years, several molecular approaches have been proposed (van Elsas et al., 2007; Kirk et al., 2004; Bloem et al., 2006; Bonomo et al., 2017; Sorensen et al., 2009) and, recently, the exploration of entire genomes present in a soil sample, metagenomics, has provided a new approach for detailed assessment (Mocali & Benedetti, 2010; Daniel, 2005; Schloss & Handelman, 2005). Metagenomics is a powerful tool for studying the soil molecular ecology, assessing the diversity of complex microbial communities, providing access to several new species, genes or novel molecules relevant for biotechnology and agricultural applications (Mocali & Benedetti, 2010).

The metagenomic approach allows to obtain useful information on the composition and genetic-physiological mechanisms of soil microbiota and their adaptation to specific environments, such as oil-contaminated soils, for a better understanding of the alterations of microbial development and biochemical activities and bioremediation processes (Mocali & Benedetti, 2010; Ahmed et al., 2018; Gomez et al., 2004; Peng et al., 2015) fundamental to re-establish soil microbial communities (Rutgers et al., 2016; Galazka et al., 2018).

Recent advances in genomics, transcriptomics and proteomics have led to increased studies on bacterial communities in contaminated soil. Genomic methods include functional bacterial fingerprinting and next-generation sequencing (NGS) of hypervariable regions, such as in 16S rRNA genes from bacteria, to determine the genetic diversity of microorganisms within a population without the need for cell culture (Galazka et al., 2018; Malla et al., 2018; Pichler et al., 2018).

In this study, different soil samples, taken ante- and post-opera the installation of an oil pipeline, were subjected to analysis of NGS to study and investigate the complex microbial biodiversity. Due to the variety of chemical-metabolic processes involved, the biodiversity of soil microorganisms plays an essential role in maintaining ecosystems in a functionally efficient state.

This study monitors the changes in the soil bacterial community’s composition in the 3 years following the construction of a crude oil flowline. The objective is to verify the possible recovery and the restoration over 3 years of the analysed territory’s initial conditions and its reuse for crops and leisure areas.

The evaluation of the displacements of the composition of the bacterial community of the soil is essential for understanding and deepening the activities and microbial dynamics in the soil, i.e. the relationships between functionality and microbial diversity, at the basis of the fundamental recovery and remediation processes. Furthermore, the aim is to identify the predominant taxa that can act as model organisms and microbial indicators of soil stress induced by the flowline construction works.

## Materials and methods

### Site description and sampling

A farmhouse located in the Basilicata region (South Italy), affected by the flowline construction works, has been the study’s sampling site. The crude oil flowline consisted of a 4-m wide excavation, 4 m deep and about 1500 m long. The flow of earthmoving vehicles involved a 40-m strip around the excavation. A forest area and a cultivated area (divided between arable land and tree crops) characterise the concerned territory’s subdivision. Figure S1 shows sampling points of the considered area. The monitoring started with soil samples collected in June and July 2013 (ante-opera monitoring) and continued during the 3 years after the flowline construction (post-opera monitoring).

Four sampling points occurred in the forest area and five in the cultivated area to assess the specific features and dynamics of microbial ecology and their evolution induced by environmental restoration processes. The monitoring program provided 2 kg of soil collected at a depth of 0–20 cm and 20–40 cm from all sampling points. A part of the sample kept in a thin layer at ambient temperature in a plastic tray was in use for physical–chemical analyses (detailed methods used and obtained data are available in Supplementary Material). The remaining part frozen at − 20 °C was useful for the subsequent DNA extraction and culture-independent microbial diversity analyses.

### 16S rDNA amplicon sequencing and sequence processing

According to the manufacturer’s protocol, soil DNA extraction was from 20 g of each soil sample, using ZR Soil Microbe DNA MicroPrep™ Kit (Zymo Research, Italy). The isolated soil DNA visualisation was possible by agarose gel (1.0%, w/v) electrophoresis running, and its quantification using Nanodrop Spectrophotometer ND-1000 (Thermo Fisher Scientific, Italy). Microbial genomic DNA extraction was in triplicate for each sample.

The extract DNA used to prepare 16S gene amplicon libraries at IGAtechnology (Udine, Italy) was according to the Illumina protocol © 16S Metagenomic Sequencing Library Preparation protocol and aiming at sequencing the V3 and V4 variable regions with primers 16S-341F 5′-CCTACGGGNGGCWGCAG-3′ and 16S-805R 5′-GACTACHVGGGTATCTAATCC-3′.

A subsequent amplification was necessary to index sequences on the sequencing cell (NexteraXT Index Kit, FC-131–1001/FC-131–1002). Finally, the libraries were sequenced in the MiSeq Illumina platform to obtain 300 bp paired reads.

At the end of the sequencing, we evaluated the reads’ quality through FASTQC. A processing was compulsory to eliminate the sequencing primers and the reads of low quality using the Trimmomatic program. An additional quality control with FASTQC was necessary.

### Bioinformatics analysis

For the metagenomic analysis and differential analysis, the GAIA pipeline was used, developed by Sequentia Biotech SL (Barcelona, Spain). This pipeline uses high quality reads to map them with BWA against the NCBI database to identify the taxonomy it belongs to and uses the low common ancestor (LCA) algorithm to classify them. Identity thresholds are applied to classify reads into operational taxonomic units (OTUs) at species, genus, family, phylum and domain levels.

LCA algorithm allowed the identification to the following percentages: identity between 0 and 70%: reads assigned at the domain level; identity between 71 and 73%: reads displayed at the phylum level; identity between 74 and 85%: reads posted at family level; correspondence between 86 and 93%: reads allocated to the genus level; and identity between 94 and 97%: reads assigned at the species level; the reads mapped to a unique species were also classified in the species reference strain to which they mapped, while the reads that did not map appeared in the report as *unknown*.

Each identified taxa could estimate the absolute abundance (reported as number of reads/pair counts) and relative abundance (reported as a percentage). The data reported as bar plots detect taxa present in each sampling point and their relative abundance. For convenience, taxa with less than 0.1% abundance in all samples were grouped as others. The samples are sorted by sampling area (forest: ABC, cultivated: DEFGH), by year (following the chronological order of sampling) and by depth, showing for each group the sampled points at 0–20 cm (1) and then those at 20–40 cm (2).

Alpha and beta diversities, calculated using Phyloseq in R, indicate the richness level (taxa number) and evenness level (taxa relative abundance) in the different soil samples. They also calculate the distance between the pairs of samples as a matrix of dissimilarity of Bray–Curtis.

Moreover, a differential abundance analysis using DESeq2, performed by GAIA, identify taxa differentially abundant in samples grouped by area and year of sampling and compared as follows: forest area 2014 vs forest area 2013, forest area in 2015 vs forest area in 2013, forest area 2016 vs forest area 2013, cultivated area 2014 vs cultivated area 2013, cultivated area 2015 vs cultivated area 2013 and cultivated area 2016 vs cultivated area 2013.

We standardised the counts resulting from the mapping of the reads to the reference database to compare different samples. Principal component analysis (PCA) was useful for studying the variation within the groups of compared samples and observing their distribution according to the relative distance.

We believed useful to construct a Volcano and an MA diagram to represent the estimated differential abundance levels.

Finally, we analysed the number of taxa for each taxonomic level as more (over-represented) or less (under-represented) abundant compared to the reference year (2013). We described their abundance as the decimal log of the fold change (logFC, i.e. the number of times the abundance has changed significantly compared to the base year 2013).

## Results and discussion

The soil disturbance created by the excavation, movement of heavy vehicles and destruction of the natural horizons of the soil and artificial reconstruction of layers, which cannot reproduce exactly the initial situation, determined a mixing of the horizons with modification of the characteristics along the soil profile. In this study, different soil samples, taken ante- and post-construction of a crude oil flowline, were analysed by next-generation sequencing (NGS) to investigate the complex microbial biodiversity that plays a vital role in maintaining ecosystems in a functionally efficient state.

**Fig. 1:**
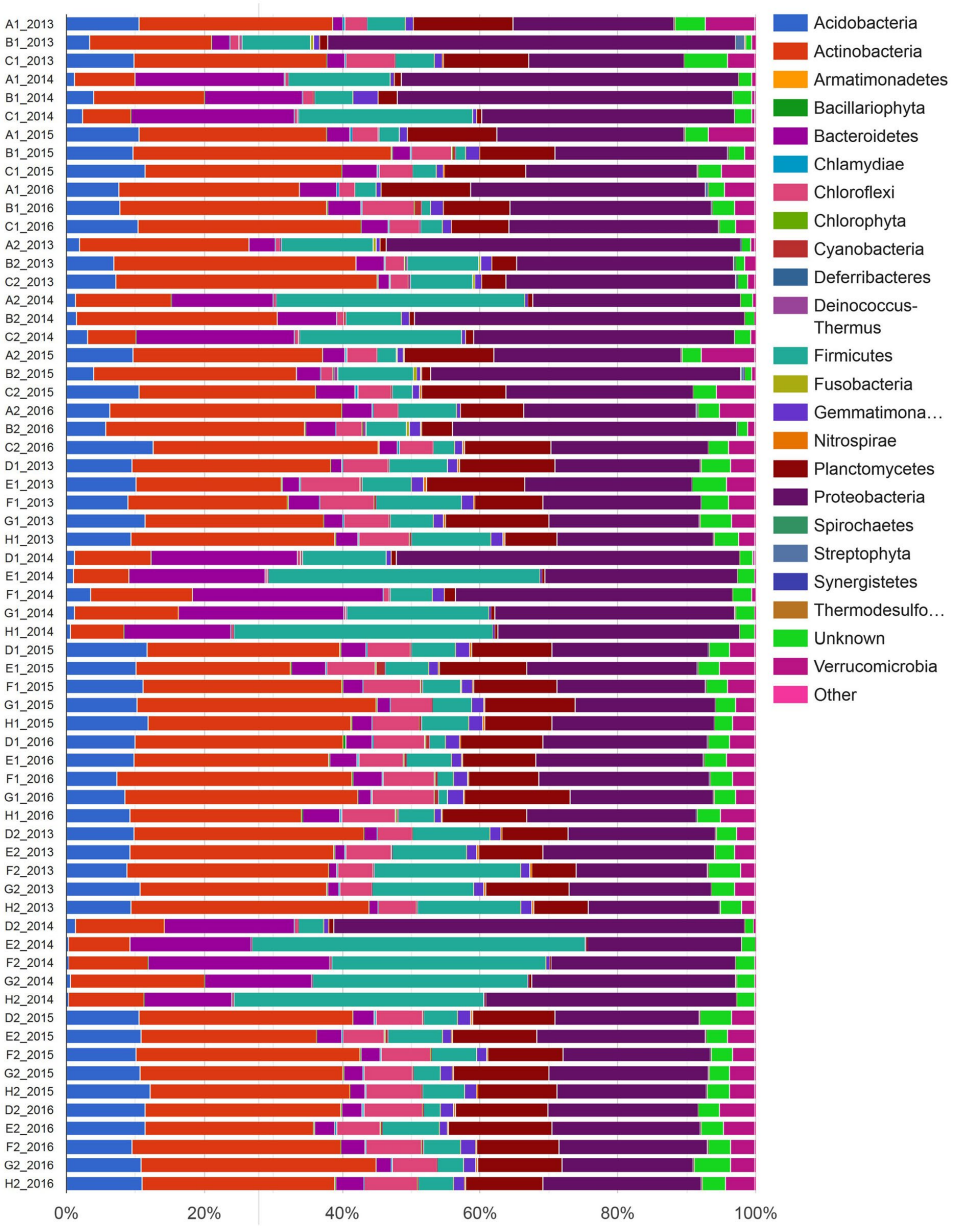
Taxa distribution in soil samples at the taxonomic level of phylum. The samples are sorted by sampling area (forest: ABC, cultivated: DEFGH), by year (following the chronological order of sampling) and by depth, showing for each group the sampling layer 0–20 cm (1) and then the layer 20–40 cm (2)

In this study, we identified a total of 56 taxa at the phylum level, 485 at the family level, 1190 at the genus level and 23,232 at the species level. Soil samples showed a different taxa distribution, especially in each of the groups collected in 2014. Considering the taxonomic level of the phylum, in Fig. 1, the overall most abundant and variable taxa appear to be Acidobacteria and Actinobacteria, which are less abundant in 2014; Bacteroidetes, which increased significantly in 2014; Chloroflexi; Firmicutes, particularly abundant on the site labelled C and agricultural soils in 2014; and Proteobacteria and Verrucomicrobia, apparently absent in the 2014 agricultural samples.

**Fig. 2:**
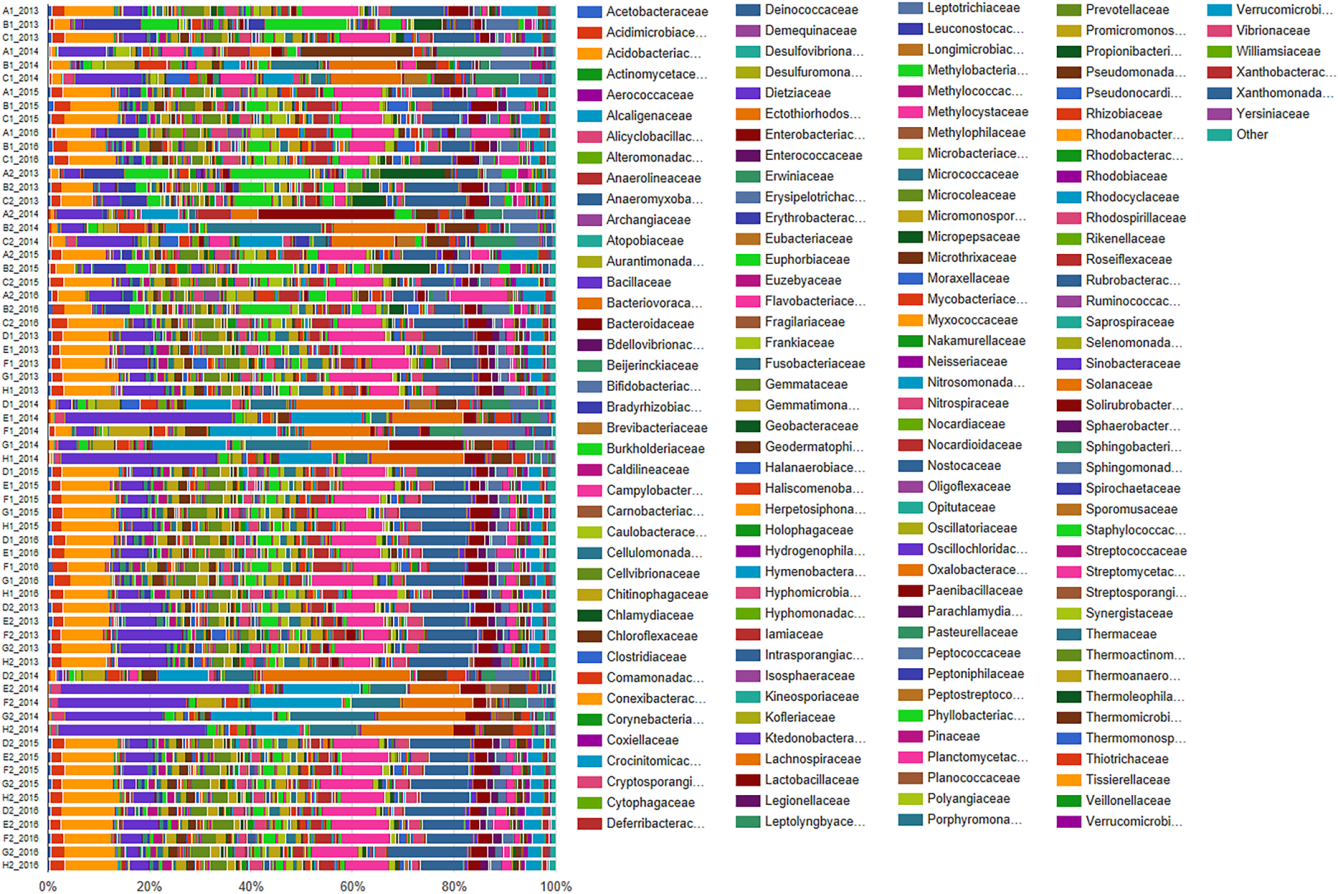
Taxa distribution in soil samples at the taxonomic level of family. The samples are sorted by sampling area (forest: ABC, cultivated: DEFGH), by year (following the chronological order of sampling) and by depth, showing for each group the sampling layer 0–20 cm (1) and then the layer 20–40 cm (2)

Moreover, regarding the family taxonomic level in Fig. 2, the abundant and variable taxa are Acidobacteriaceae, Bacillaceae, Hymenobacteriaceae, Micrococcaceae, Oxalobacteriaceae, Paenibacillaceae, Planctomycetaceae, Pseudomonadaceae, Propionibacteriaceae and Rubrobacteriaceae.

**Fig. 3:**
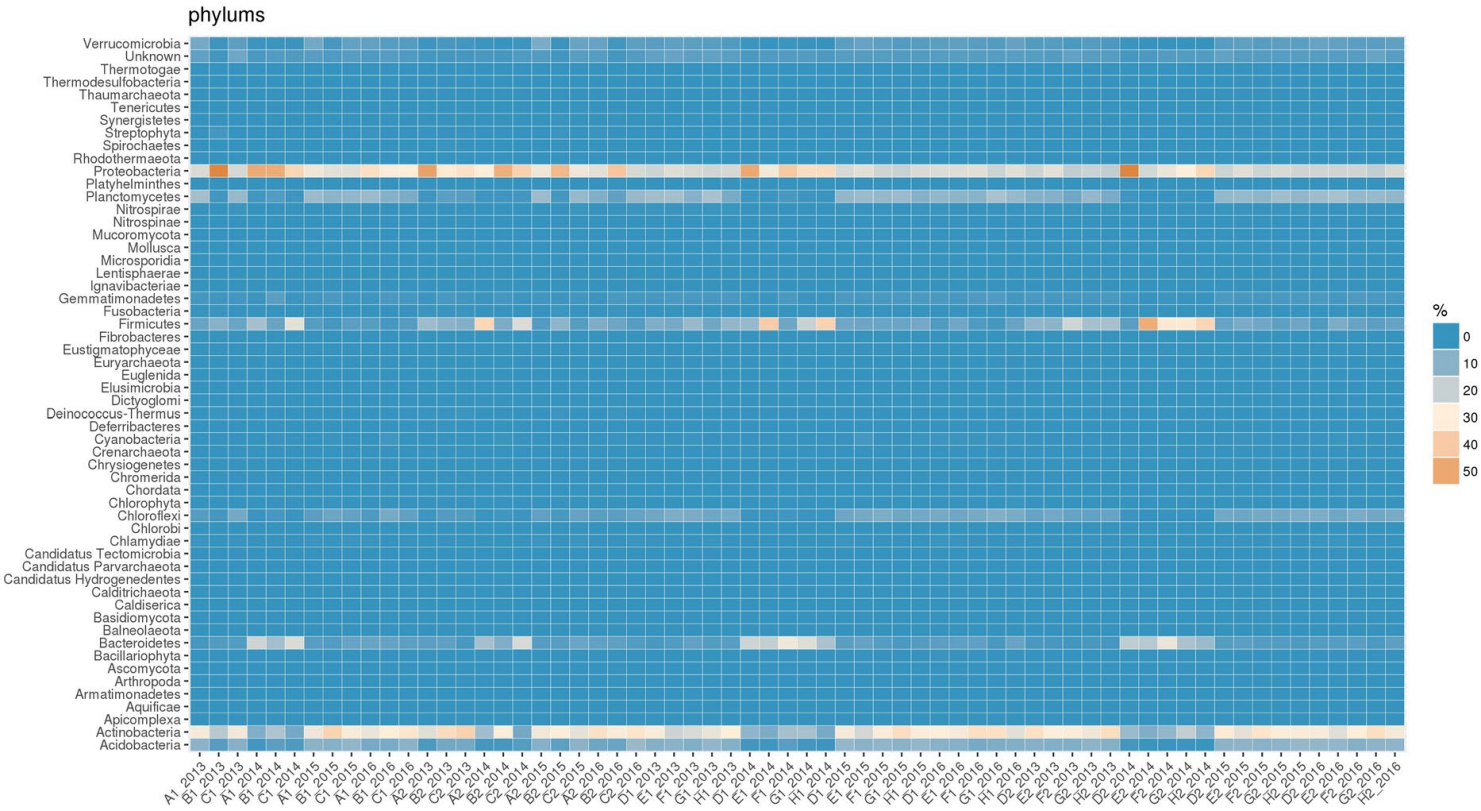
Heatmap for data visualisation at the taxonomic level of phylum; abundance blocks of different taxa in the sampling sites during the 3 years following the flowline’s construction

**Fig. 4:**
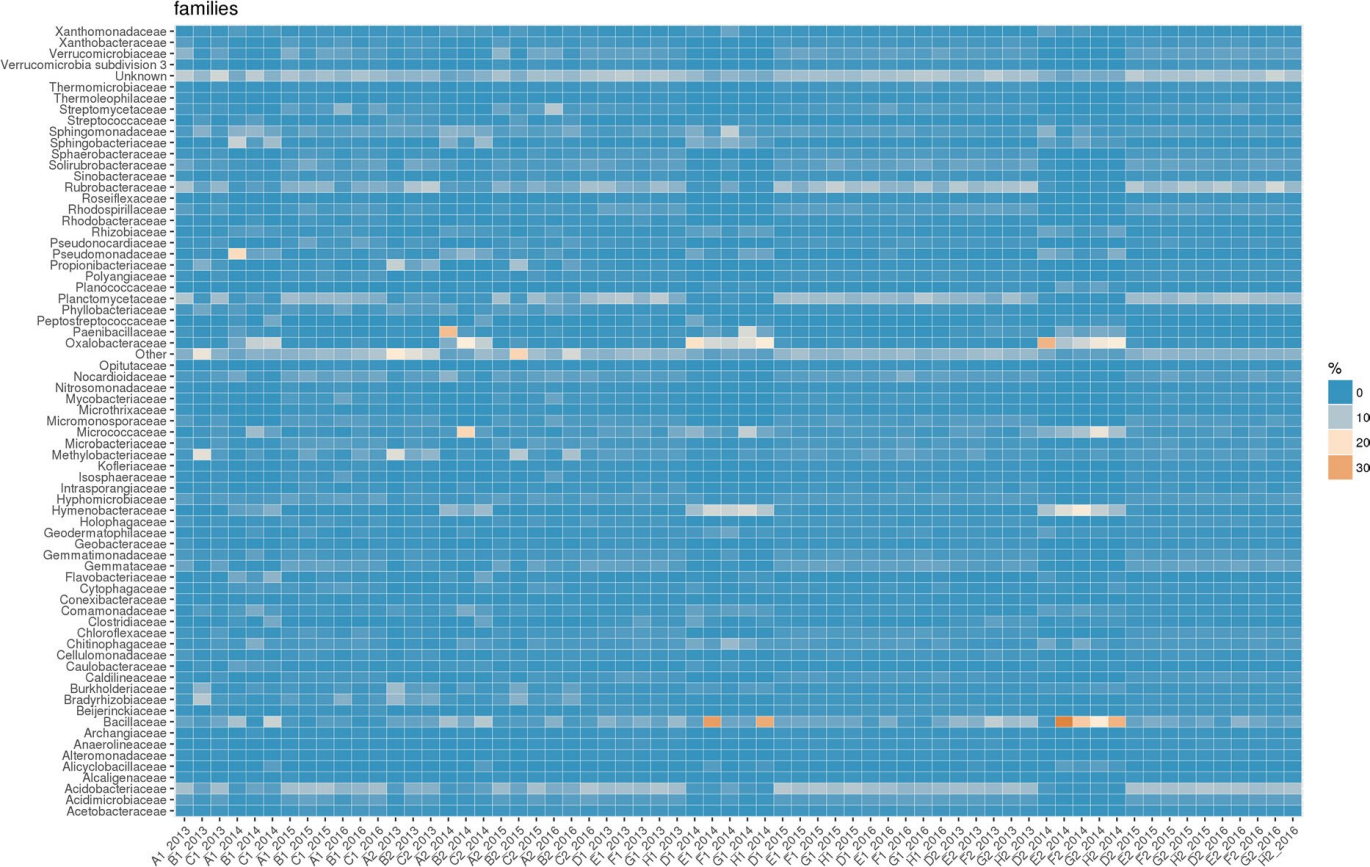
Heatmap for data visualisation at the taxonomic level of family; abundance blocks of different taxa in the sampling sites during the 3 years following the flowline’s construction

The heatmaps, built for easy and immediate visualisation of data, presented the best results at the taxonomic levels of phylum and family. Figures 3 and 4 clearly show the abundance blocks of different taxa in sampling sites during the three years following the flowline’s construction, suggesting changes, i.e. biodiversity.

The results obtained agree with previous works that showed differences in soil microbial community structure and taxonomic composition due to different anthropic insults (Ahmed et al., 2018; Galazka et al., 2018; Carbonetto et al., 2014; Liu et al., 2017; Klimek et al., 2016). We observed significant alterations of microbiological dynamics following the flowline construction compared to the reference year 2013 (ante-opera monitoring) suggesting their evolution induced by environmental restoration processes. Also, changes were detected in the post-opera years, highlighting a specific adaptation to the altered conditions by select groups and types of bacteria. Changes in bacterial communities observed in post-opera analyses demonstrated modifications of the genetic and physiological bacterial response to face stress. Bacterial communities implemented different mechanisms to survive and adapt to the modified conditions of the soil environment.

As shown in Tables S1 and S2, the extensive soil disturbance due to the flowline construction inevitably caused direct and indirect changes in soil characteristics. The forest area has undergone a substantial evolution with a noteworthy diversification of chemical-physical parameters between the different sampling points. The chemical-physical properties of the examined soils were initially almost uniform but showed significant annual variations. The phenomenon indicates aggregation processes favouring organo-mineral associations in an environment with good microbial biomass activity that can suggest the development of widespread root systems.

The pH values tend to evolve towards greater alkalinity, which is also identifiable in the CEC values that are all higher than the initial ones, even when reducing the free organic matter.

We hypothesise that, in addition to the climatic factors and rainfall, always abundant in the area (data not shown), the soil’s movement first, and the development of grassy vegetation then, played a fundamental role in the observed evolutionary process. The reduction of organic carbon is well observable together with a similar decrease in total nitrogen, exchangeable potassium and assimilable phosphorus.

The values of concentrations found for the heavy hydrocarbons (from C10 to C40) were generally under the limit of detection attributable to the chromatographic method used (5 mg/kg of dry matter). In 2014, for both the forest area and the cultivated area, values unusually higher of hydrocarbons were recorded in either the superficial- and deep-sampled soil layers (16–32 mg/kg dm). This event suggests a small and momentary leak in the oil pipeline that was operative at the end of 2013. In 2015, a weird value appeared only in a deep sample collected from the agricultural area (36 mg/kg dm). Such a recovery process occurs only when different microbial species can transform the hydrocarbons into energy, attending a natural bioremediation/biodegradation activity and restoring the initial soil conditions.

Since microbiological diversity changes occurred in the samples collected after the year 2013, a comparative analysis was necessary to identify taxonomies whose abundance is significantly changing over time. Therefore, we performed a differential abundance analysis using DESeq2 by GAIA to identify taxa differentially represented.

We evaluated the abundance of taxa by comparing the forest area sampling in 2014 and the selection of 2013. The Principal component analysis in Fig. S2a showed a variability of the 2014 samples more significantly than the 2013 samples at the phylum taxonomic level, explaining the new biological differentiation between the two sampling years. The analysis of the differences groups the principal component 1 (*X*-axis) quite differently over the 2 years, while the 2013 samples clustered in a more homogeneous group. The main component 1 explains 75% of the total variation. The method adopted recognised 56 phyla in this group of samples; 14 of these are differentially abundant in 2014 compared to the reference year (2013). In particular, eight phyla seem over-represented, i.e. more abundant in 2014 than 2013, and 6 under-represented, i.e. they showed a lower abundance in 2014.

At the family taxonomic level, the PCA showed an even more significant variability among the 2014 samples than those of 2013 regarding biological diversity. The main component 2, accounting for 22% variability, collects most of the diversity of the 2014 collected soils, except for site A at a depth of 20–40 m, which is very far from the others along the X-axis in Fig. S2b. This group of samples identify 485 taxonomic families. The comparative analysis highlighted that 126 families are differentially present in 2014 compared to 2013. In particular, 65 increased quantitatively, and 59 significantly decreased in 2014. Table 1 shows the significant taxa under and over-represented, with the relative value of logFC.

**Table 1:** Taxa identified at taxonomic level of family in soil samples of forest area collected in the 3 years following the installation of the oil pipeline (2014, 2015, 2016). Taxa are reported as more (over-represented) or less (under-represented) abundant compared to the reference year (2013), and their abundance has been described as the decimal logarithm of the fold change (log FC, i.e., the number of times that the abundance is significantly changed compared to the reference)

Forest area											
**2014**				**2015**				**2016**			
**OTUs** **Under-represented**	**LogFC**	**OTUs** **Over-represented**	**LogFC**	**OTUs** **Under-represented**	**LogFC**	**OTUs** **Over-represented**	**LogFC**	**OTUs** **Under-represented**	**LogFC**	**OTUs** **Over-represented**	**LogFC**
Psychromonadaceae	− 6.06327276	Hymenobacteraceae	7.888895127	Brevibacteriaceae	− 3.16186973	Cyclobacteriaceae	4.66440479	Psychromonadaceae	− 6.48599001	Microcoleaceae	6.45362388
Neisseriaceae	− 5.62965830	Gloeobacteraceae	7.888878258	Bacillaceae	− 1.28151207	Cellvibrionaceae	4.55881690	Leuconostocaceae	− 5.24088706	Pinaceae	4.10986600
Propionibacteriaceae	− 5.46734860	Alicyclobacillaceae	6.370363515			Glycomycetaceae	3.55458094	Hydrogenophilaceae	− 3.40865156	Williamsiaceae	4.06749720
Leptotrichiaceae	− 5.36114366	Sphingobacteriaceae	6.314451742			Williamsiaceae	3.23929522	Tissierellaceae	− 3.37275403	Marinifilaceae	3.90363172
Sphagnaceae	− 5.32059292	Oxalobacteraceae	6.150946723			Hyphomonadaceae	2.36438174	Erysipelotrichaceae	− 3.00831050	Cellvibrionaceae	3.60524439
Pasteurellaceae	− 5.04593850	Erwiniaceae	5.652816453			Nocardiaceae	1.86393770	Propionibacteriaceae	− 2.78856420	Hyphomonadaceae	3.06740116
Ktedonobacteraceae	− 4.92775787	Cellvibrionaceae	5.593468041			Cytophagaceae	1.82351153	Clostridiales Family XIII. Incertae Sedis	− 2.46836969	Cryomorphaceae	2.89053543
Prevotellaceae	− 4.91290704	Cyclobacteriaceae	5.427717144					Alicyclobacillaceae	− 2.30524104	Isosphaeraceae	2.69205937
Akkermansiaceae	− 4.87990999	Hafniaceae	4.930928318					Peptococcaceae	− 1.89791808	Flammeovirgaceae	2.63898185
Dietziaceae	− 4.61547249	Flavobacteriaceae	4.924584859					Syntrophomonadaceae	− 1.82125746	Nocardiaceae	2.47110072
Peptoniphilaceae	− 4.45037741	Pseudomonadaceae	4.783010581					Ruminococcaceae	− 1.77561340	Mycobacteriaceae	2.41353519
Staphylococcaceae	− 4.20204296	Rhabdochlamydiaceae	4.680061978					Sporomusaceae	− 1.60058263	Streptomycetaceae	2.32343025
Catenulisporaceae	− 4.07816386	Holosporaceae	4.509087642					Clostridiaceae	− 1.53632553	Cytophagaceae	2.31584838
Ferritrophicaceae	− 3.91210126	Thermonemataceae	4.432242017					Thermodesulfobacteriaceae	− 1.50303662	Bdellovibrionaceae	2.04844576
Bacteroidaceae	− 3.83778397	Peptostreptococcaceae	4.423008784					Thermoleophilaceae	− 1.14555465	Rhizobiaceae	1.94187171
Veillonellaceae	− 3.83001210	Francisellaceae	4.216568515					Thermoanaerobacteraceae	− 0.99888133	Nannocystaceae	1.84166128
Selenomonadaceae	− 3.78102334	Micrococcaceae	4.049633374							Rhodanobacteraceae	1.70150532
Actinospicaceae	− 3.75944153	Marinifilaceae	4.04475163							Sphingobacteriaceae	1.66053291
Lactobacillaceae	− 3.73908507	Bernardetiaceae	3.869712263							Nocardioidaceae	1.16464077
Corynebacteriaceae	− 3.63505712	Flatidae	3.84154994							Acetobacteraceae	0.75039264
Frankiaceae	− 3.61926727	Rhizobiaceae	3.84072105								
Fusobacteriaceae	− 3.61836305	Clostridiaceae	3.595571114								
Aerococcaceae	− 3.60792038	Haliscomenobacteraceae	3.474238951								
Nakamurellaceae	− 3.40746280	Oscillatoriaceae	3.472564336								
Porphyromonadaceae	− 3.22918041	Clostridiales Family XII. Incertae Sedis	3.400473793								
Pseudonocardiaceae	− 3.18042590	Oligoflexaceae	3.302744387								
Roseiflexaceae	− 3.00834859	Deinococcaceae	3.300654812								
Thermaceae	− 2.89337022	Halothiobacillaceae	3.228044845								
Methylobacteriaceae	− 2.80724413	Bacteriovoracaceae	3.045339681								
Anaerolineaceae	− 2.66960077	Bdellovibrionaceae	2.924723969								
Tissierellaceae	− 2.62661255	Cytophagaceae	2.923583399								
Hydrogenophilaceae	− 2.46663879	Lewinellaceae	2.910131074								
Micromonosporaceae	− 2.43512398	Crocinitomicaceae	2.891005883								
Isosphaeraceae	− 2.39201177	Enterobacteriaceae	2.861893096								
Atopobiaceae	− 2.28180376	Caulobacteraceae	2.826834131								
Jiangellaceae	− 2.17383769	Chitinophagaceae	2.670955823								
Anaeromyxobacteraceae	− 2.16691247	Coleofasciculaceae	2.66427167								
Verrucomicrobia Subdivision 3	− 2.15842037	Beutenbergiaceae	2.611973333								
Syntrophomonadaceae	− 2.15170610	Sporolactobacillaceae	2.580366721								
Thermomonosporaceae	− 2.15036281	Bacillaceae	2.550785896								
Alteromonadaceae	− 2.07875561	Amoebophilaceae	2.537815722								
Actinomycetaceae	− 2.07430734	Xanthomonadaceae	2.503417977								
Euzebyaceae	− 2.05095497	Sutterellaceae	2.40189153								
Streptosporangiaceae	− 1.94537245	Hyphomonadaceae	2.400816655								
Geobacteraceae	− 1.93234036	Pectobacteriaceae	2.328801718								
Solibacteraceae	− 1.92872557	Comamonadaceae	2.25932381								
Bradyrhizobiaceae	− 1.92604737	Planococcaceae	2.177317534								
Thermoanaerobacterales Family III. Incertas Sedis	− 1.75416278	Acholeplasmataceae	2.146014836								
Demequinaceae	− 1.73685700	Flammeovirgaceae	2.094420124								
Nocardiopsaceae	− 1.73315518	Mycoplasmataceae	2.092104113								
Microthrixaceae	− 1.55348794	Morganellaceae	2.066591591								
Thermodesulfobacteriaceae	− 1.50559896	Shewanellaceae	2.049714384								
Sphaerobacteraceae	− 1.50133850	Gallionellaceae	2.041974323								
Cellulomonadaceae	− 1.29261153	Nocardioidaceae	1.931478609								
Thermoanaerobacteraceae	− 1.25679848	Sphingomonadaceae	1.919595269								
Caldilineaceae	− 1.22947297	Legionellaceae	1.872108254								
Xanthobacteraceae	− 1.22939589	Brucellaceae	1.808183289								
Coriobacteriaceae	− 1.17013321	Marinilabiliaceae	1.741838928								
Rubrobacteraceae	− 1.03537168	Geodermatophilaceae	1.536473395								
		Candidatus Midichloriaceae	1.448790415								
		Helicobacteraceae	1.424805881								
		Chromobacteriaceae	1.424495355								
		Halomonadaceae	1.408695854								
		Rhodocyclaceae	1.296696518								
		Chromatiaceae	0.922741716								

The relative abundances expressed in terms of logFC covered a fairly wide range (− 6.06 ≤ logFC $\ge$ 7.88). The families with the most differential relative abundance are Psychromonadaceae, Neisseriaceae, Propionibacteriaceae, Leptotrichiaceae, Sphagnaceae and Pasteurellaceae.

As the comparison forest area 2015 vs forest area 2013, the assessment of identified taxa abundance shown in PCA presented a lower variability between 2013 and 2015 than the highlighted for the year 2014 in Fig. S3.

At the phylum level classification, there were no changes in the abundance of the 56 taxa identified at this taxonomic level. Of the 485 families detected, only nine were differentially abundant and in particular 7 (especially Cyclobacteriaceae and Cellvibrionaceae) over-represented and 2 (Brevibacteriaceae and Bacillaceae) under-represented, with variable fold changes in logarithmic scale between − 3.16 and 4.66 (Table 1).

Comparing the forest area 2016 to forest area 2013, the PCA does not significantly distance biological diversity at the phylum level. A greater biological diversity exists within the forest area in 2013, primarily for site B at depth 0–20 cm (Fig. S4a and b).

A total of 485 taxonomic families were identified, of which 20 are significantly more rich (such as Microcoleaceae, Pinaceae and Williamsiaceae) and 16 less abundant (such as Psychromonadaceae and Leuconostocaceae) in 2016 compared to the reference year, with variable fold changes between − 6.48 and 6.45 on a logarithmic scale (Table 1).

Comparing the microbial composition of samples collected in the cultivated area during 2014 to the area sampled in the 2013, at the phylum level, PCA showed a substantial variability among the samples of 2014, especially along the principal component 1, which explains more than 87% of variability, while the samples taken in 2013 clustered together, due to a minimal variability (Fig. S5a). These samples identified 56 phyla, of which 33 showed differential abundance in 2014; in particular, 14 are more abundant and 19 less abundant in 2014. At the taxonomic family level, the PCA still showed more significant variability among the 2014 samples (Fig. S5b).

The families identified were 485, but only 260 were differentially abundant in 2014, 123 over-represented and 137 under-represented (Table 2). Table 2 shows the identification of these taxa and their relative abundance.

**Table 2:** Taxa identified at taxonomic level of family in soil samples of cultivated area collected in the 3 years following the installation of the oil pipeline (2014, 2015, 2016). Taxa are reported as more (over-represented) or less (under-represented) abundant compared to the reference year (2013), and their abundance has been described as the decimal logarithm of the fold change (log FC, i.e., the number of times that the abundance is significantly changed compared to the reference)

				**Cultivated area**							
**2014**				**2015**				**2016**			
**OTUs** **Under-represented**	**LogFC**	**OTUs** **Over-represented**	**LogFC**	**OTUs** **Under-represented**	**LogFC**	**OTUs** **Over-represented**	**LogFC**	**OTUs** **Under-represented**	**LogFC**	**OTUs** **Over-represented**	**LogFC**
Ktedonobacteraceae	− − 5.67745313	Flatidae	7.76578242	Ktedonobacteraceae	− 5.67745313	Williamsiaceae	4.01955009	Methanosarcinaceae	− 5.31935895	Stephanopyxidaceae	5.38043382
Methanosarcinaceae	− 5.44262133	Hymenobacteraceae	7.38339489	Methanosarcinaceae	− 5.44262133	Marinifilaceae	2.96457849	Erysipelotrichaceae	− 4.57562628	Coccomyxaceae	4.90260604
Nitrososphaeraceae	− 5.32871599	Oxalobacteraceae	7.18752404	Nitrososphaeraceae	− 5.32871599	Tolypothrichaceae	2.96070390	Defluviitaleaceae	− 4.39007583	Fragilariaceae	4.52946383
Nostocaceae	− 4.56828357	Erwiniaceae	6.68868395	Nostocaceae	− 4.56828357	Aeromonadaceae	2.95465374	Prolixibacteraceae	− 4.19559556	Williamsiaceae	4.33771564
Isosphaeraceae	− 4.46356073	Leuconostocaceae	6.19628286	Isosphaeraceae	− 4.46356073	Procabacteriaceae	2.94911728	Peptostreptococcaceae	− 3.68946772	Chattonellaceae	4.02160377
Roseiflexaceae	− 4.32432687	Pseudomonadaceae	6.11934693	Roseiflexaceae	− 4.32432687	Microcoleaceae	2.91569484	Methanomicrobiaceae	− 3.35860031	Psilotaceae	3.97180420
Bryobacteraceae	− 4.26980877	Sphingobacteriaceae	5.67694324	Bryobacteraceae	− 4.26980877	Gomontiellaceae	2.86574304	Alicyclobacillaceae	− 3.26837245	Tolypothrichaceae	3.83723923
Ferritrophicaceae	− 4.23793450	Bacteriovoracaceae	5.12739743	Ferritrophicaceae	− 4.23793450	Methanomassiliicoccaceae	2.70926163	Thermoactinomycetaceae	− 3.26155126	Rhizosoleniaceae	3.62551973
Phycisphaeraceae	− 4.22776437	Chrysiogenaceae	5.11287505	Phycisphaeraceae	− 4.22776437	Psilotaceae	2.61763482	Symbiobacteriaceae	− 2.88048028	Methanomassiliicoccaceae	3.50568603
Caldicoprobacteraceae	− 4.18822197	Haliscomenobacteraceae	5.00249325	Caldicoprobacteraceae	− 4.18822197	Fragilariaceae	2.43314861	Limnochordaceae	− 2.86272884	Osmundaceae	3.48348440
Sphagnaceae	− 4.18586886	Gloeobacteraceae	4.84284670	Sphagnaceae	− 4.18586886	Sphagnaceae	2.37594661	Christensenellaceae	− 2.72530159	Erwiniaceae	3.30937089
Microcoleaceae	− 4.09737664	Alicyclobacillaceae	4.81326179	Microcoleaceae	− 4.09737664	Pedinomonadaceae	2.09603026	Clostridiaceae	− 2.72374182	Budviciaceae	3.29364593
Anaerolineaceae	− 3.95803400	Marinifilaceae	4.76134959	Anaerolineaceae	− 3.95803400	Cellvibrionaceae	2.07359064	Halanaerobiaceae	− 2.69326827	Rhabdochlamydiaceae	3.09654584
Alteromonadaceae	− 3.93290202	Rhizobiaceae	4.51577167	Alteromonadaceae	− 3.93290202	Bacillariaceae	1.91300857	Lachnospiraceae	− 2.67410514	Marinifilaceae	3.07077724
Thermosporotrichaceae	− 3.77868030	Streptococcaceae	4.47617291	Thermosporotrichaceae	− 3.77868030	Saccharospirillaceae	1.76994976	Catabacteriaceae	− 2.55650515	Coleochaetaceae	3.06162711
Dictyoglomaceae	− 3.74176509	Flavobacteriaceae	4.35733620	Dictyoglomaceae	− 3.74176509	Coscinodiscaceae	1.48642667	Tissierellaceae	− 2.55167934	Bacillariaceae	2.98967704
Symbiobacteriaceae	− 3.63980065	Bernardetiaceae	4.27594649	Symbiobacteriaceae	− 3.63980065	Coxiellaceae	1.33004998	Gracilibacteraceae	− 2.47943139	Solanaceae	2.83497472
Halobacteriaceae	− 3.58178672	Blattabacteriaceae	4.22426817	Halobacteriaceae	− 3.58178672	Bdellovibrionaceae	0.98520390	Sporomusaceae	− 2.44106395	Spirulinaceae	2.82408396
Fragilariaceae	− 3.55799454	Enterobacteriaceae	4.15991448	Fragilariaceae	− 3.55799454	Hymenobacteraceae	0.98445737	Paenibacillaceae	− 2.42108568	Microcoleaceae	2.80554994
Sphaerobacteraceae	− 3.50581327	Paenibacillaceae	4.01480835	Sphaerobacteraceae	− 3.50581327	Bacteriovoracaceae	0.98243685	Ruminococcaceae	− 2.39603961	Cryptomonadaceae	2.78311748
Anaeromyxobacteraceae	− 3.48395979	Xanthomonadaceae	3.89235930	Anaeromyxobacteraceae	− 3.48395979	Mycobacteriaceae	0.86143516	Clostridiales Family XIII. Incertae Sedis	− 2.23523981	Eupodiscaceae	2.68623752
Thermomicrobiaceae	− 3.45966028	Thermonemataceae	3.78414324	Thermomicrobiaceae	− 3.45966028	Tsukamurellaceae	0.74932255	Syntrophomonadaceae	− 2.11718618	Fabaceae	2.66817000
Nakamurellaceae	− 3.41477526	Salinisphaeraceae	3.74300672	Nakamurellaceae	− 3.41477526	Rhodanobacteraceae	0.71654194	Planococcaceae	− 2.10168210	Saccharospirillaceae	2.44924840
Nitrospiraceae	− 3.30250817	Gallionellaceae	3.68695066	Nitrospiraceae	− 3.30250817	Xanthomonadaceae	0.65687181	Caldicoprobacteraceae	− 2.08003555	Marsupiomonadaceae	2.40298751
Caldilineaceae	− 3.24559076	Comamonadaceae	3.66293245	Caldilineaceae	− 3.24559076	Rhodobiaceae	0.51152872	Peptococcaceae	− 2.00121007	Hydrodictyaceae	2.39901611
Parachlamydiaceae	− 3.20139726	Saccharospirillaceae	3.63085555	Parachlamydiaceae	− 3.20139726	Kofleriaceae	0.50841064	Glycomycetaceae	− 1.98310888	Ferrovaceae	2.36116396
Bacillariaceae	− 3.20135641	Spirillaceae	3.62891151	Bacillariaceae	− 3.20135641	Alcaligenaceae	0.50678895	Carnobacteriaceae	− 1.90011464	Aeromonadaceae	2.33008415
Verrucomicrobia subdivision 3	− 3.18555160	Hafniaceae	3.59614240	Verrucomicrobia subdivision 3	− 3.18555160	Streptococcaceae	0.42319004	Sporolactobacillaceae	− 1.86154581	Coscinodiscaceae	2.30614838
Syntrophomonadaceae	− 3.18524639	Planococcaceae	3.58739671	Syntrophomonadaceae	− 3.18524639	Polyangiaceae	0.24543152	Pasteuriaceae	− 1.73815894	Oscillatoriaceae	2.28853040
Limnochordaceae	− 3.11016522	Caulobacteraceae	3.56142798	Limnochordaceae	− 3.11016522			Proteinivoraceae	− 1.50700072	Yersiniaceae	2.27841071
Planctomycetaceae	− 3.10754247	Micrococcaceae	3.53688746	Planctomycetaceae	− 3.10754247			Selenomonadaceae	− 1.50649496	Hafniaceae	2.14101806
Skeletonemataceae	− 3.10696431	Lewinellaceae	3.53507011	Skeletonemataceae	− 3.10696431			Veillonellaceae	− 1.50253717	Chlorodendraceae	2.14018501
Methylacidiphilaceae	− 3.10296088	Pelagibacteraceae	3.52359858	Methylacidiphilaceae	− 3.10296088			Peptoniphilaceae	− 1.49857967	Sphagnaceae	2.08850302
Catabacteriaceae	− 3.08665594	Coxiellaceae	3.37395102	Catabacteriaceae	− 3.08665594			Natranaerobiaceae	− 1.40193707	Pedinomonadaceae	1.96875682
Micromonosporaceae	− 3.06998039	Bacillaceae	3.34210402	Micromonosporaceae	− 3.06998039			Desulfovibrionaceae	− 1.34611803	Chroomonadaceae	1.95205548
Thermoactinomycetaceae	− 3.03179398	Moraxellaceae	3.31163562	Thermoactinomycetaceae	− 3.03179398			Deferribacteraceae	− 1.33505309	Cryomorphaceae	1.90027074
Nannocystaceae	− 2.97083909	Microscillaceae	3.28160234	Nannocystaceae	− 2.97083909			Clostridiales Family XII. Incertae Sedis	− 1.20595276	Coxiellaceae	1.89654267
Nitrospinaceae	− 2.96199400	Spiroplasmataceae	3.25697567	Nitrospinaceae	− 2.96199400			Eubacteriaceae	− 1.18499417	Cellvibrionaceae	1.74615741
Dehalococcoidaceae	− 2.95502042	Ferrovaceae	3.24167241	Dehalococcoidaceae	− 2.95502042			Spirochaetaceae	− 1.18307685	Haliscomenobacteraceae	1.64706914
Erysipelotrichaceae	− 2.93524756	Carnobacteriaceae	3.18119280	Erysipelotrichaceae	− 2.93524756			Bacillaceae	− 1.16748553	Crocinitomicaceae	1.64275691
Pseudonocardiaceae	− 2.90751778	Burkholderiaceae	3.15156525	Pseudonocardiaceae	− 2.90751778			Staphylococcaceae	− 1.14879961	Skeletonemataceae	1.62643143
Syntrophobacteraceae	− 2.90696050	Cellvibrionaceae	3.12002312	Syntrophobacteraceae	− 2.90696050			Anaeromyxobacteraceae	− 1.14854420	Halieaceae	1.60674561
Aphanizomenonaceae	− 2.88561826	Peptostreptococcaceae	3.10291319	Aphanizomenonaceae	− 2.88561826			Desulfobulbaceae	− 1.13977624	Oleiphilaceae	1.49671742
Actinospicaceae	− 2.86252695	Chitinophagaceae	3.08378222	Actinospicaceae	− 2.86252695			Syntrophobacteraceae	− 1.03173183	Pseudomonadaceae	1.43046964
Leptolyngbyaceae	− 2.85681994	Bdellovibrionaceae	3.06679976	Leptolyngbyaceae	− 2.85681994			Vibrionaceae	− 1.01500472	Aurantimonadaceae	1.39260060
Verrucomicrobiaceae	− 2.85137777	Nautiliaceae	3.06153199	Verrucomicrobiaceae	− 2.85137777			Amoebophilaceae	− 0.96514819	Enterobacteriaceae	1.38222039
Symphyonemataceae	− 2.84666397	Lactobacillaceae	3.03140517	Symphyonemataceae	− 2.84666397			Fusobacteriaceae	− 0.93690835	Nocardiaceae	1.23706386
Tissierellaceae	− 2.71001416	Holosporaceae	3.02847238	Tissierellaceae	− 2.71001416			Solibacteraceae	− 0.93332625	Saprospiraceae	1.12781973
Defluviitaleaceae	− 2.69112120	Francisellaceae	3.02816441	Defluviitaleaceae	− 2.69112120			Nocardiopsaceae	− 0.92656799	Flavobacteriaceae	1.11413256
Halanaerobiaceae	− 2.68910720	Microcystaceae	2.98349296	Halanaerobiaceae	− 2.68910720			Pasteurellaceae	− 0.91948669	Bacteriovoracaceae	1.09471151
Prolixibacteraceae	− 2.68324147	Deinococcaceae	2.89544721	Prolixibacteraceae	− 2.68324147			Micrococcaceae	− 0.90405821	Tsukamurellaceae	1.08685528
Euzebyaceae	− 2.67674888	Aurantimonadaceae	2.88463964	Euzebyaceae	− 2.67674888			Streptosporangiaceae	− 0.89739544	Opitutaceae	1.07185497
Methanomicrobiaceae	− 2.67492753	Dietziaceae	2.71737041	Methanomicrobiaceae	− 2.67492753			Nitrospiraceae	− 0.84356731	Mycobacteriaceae	1.05027631
Ectothiorhodospiraceae	− 2.65079089	Geodermatophilaceae	2.62195731	Ectothiorhodospiraceae	− 2.65079089			Coriobacteriaceae	− 0.80400928	Alcanivoracaceae	1.03265593
Sporomusaceae	− 2.62434262	Peptoniphilaceae	2.56700080	Sporomusaceae	− 2.62434262			Thermoanaerobacteraceae	− 0.78699607	Streptococcaceae	0.95833515
Pedinomonadaceae	− 2.60620601	Succinivibrionaceae	2.53349360	Pedinomonadaceae	− 2.60620601			Methylococcaceae	− 0.74039180	Rhodanobacteraceae	0.92526458
Acidothermaceae	− 2.60072278	Coleofasciculaceae	2.49792084	Acidothermaceae	− 2.60072278			Rickettsiaceae	− 0.68670236	Xanthomonadaceae	0.89655994
Streptosporangiaceae	− 2.55798714	Sporolactobacillaceae	2.46114919	Streptosporangiaceae	− 2.55798714			Bifidobacteriaceae	− 0.66808222	Marinilabiliaceae	0.82817780
Nocardiopsaceae	− 2.52225945	Pectobacteriaceae	2.44207059	Nocardiopsaceae	− 2.52225945			Eggerthellaceae	− 0.62681822	Syntrophaceae	0.82109719
Streptomycetaceae	− 2.51011721	Sulfuricellaceae	2.43616681	Streptomycetaceae	− 2.51011721			Anaplasmataceae	− 0.60482501	Chitinophagaceae	0.79957352
Microthrixaceae	− 2.50955550	Brucellaceae	2.38308897	Microthrixaceae	− 2.50955550			Desulfuromonadaceae	− 0.59847378	Sphingobacteriaceae	0.78035668
Geobacteraceae	− 2.47673303	Sphingomonadaceae	2.37363918	Geobacteraceae	− 2.47673303			Rhodospirillaceae	− 0.58544202	Bdellovibrionaceae	0.75094070
Gemmataceae	− 2.46396195	Clostridiales Family XII. Incertae Sedis	2.34438602	Gemmataceae	− 2.46396195			Actinomycetaceae	− 0.44754521	Cytophagaceae	0.71030918
Chthonomonadaceae	− 2.42138407	Sutterellaceae	2.33330848	Chthonomonadaceae	− 2.42138407					Streptomycetaceae	0.70346092
Fibrobacteraceae	− 2.41641796	Colwelliaceae	2.33092945	Fibrobacteraceae	− 2.41641796					Micropepsaceae	0.68761417
Gracilibacteraceae	− 2.33667880	Brevinemataceae	2.32490873	Gracilibacteraceae	− 2.33667880					Flammeovirgaceae	0.66433495
Solibacteraceae	− 2.29750099	Cytophagaceae	2.31113175	Solibacteraceae	− 2.29750099					Kofleriaceae	0.65143113
Chloroflexaceae	− 2.28934544	Rikenellaceae	2.30219286	Chloroflexaceae	− 2.28934544					Legionellaceae	0.64298519
Thermomonosporaceae	− 2.26702361	Morganellaceae	2.27391185	Thermomonosporaceae	− 2.26702361					Verrucomicrobia subdivision 3	0.59165552
Xanthobacteraceae	− 2.25283561	Prevotellaceae	2.25732617	Xanthobacteraceae	− 2.25283561					Rhodobiaceae	0.58624837
Opitutaceae	− 2.20643799	Acholeplasmataceae	2.24636097	Opitutaceae	− 2.20643799					Caldilineaceae	0.57158509
Frankiaceae	− 2.18882628	Mycoplasmataceae	2.23900101	Frankiaceae	− 2.18882628					Alcaligenaceae	0.53941169
Coriobacteriaceae	− 2.16558129	Clostridiales Family XIII. Incertae Sedis	2.21725001	Coriobacteriaceae	− 2.16558129					Gemmataceae	0.51721228
Halieaceae	− 2.14721626	Legionellaceae	2.21086924	Halieaceae	− 2.14721626					Phyllobacteriaceae	0.48772819
Thermoanaerobacterales Family III. Incertae Sedis	− 2.11155189	Chromobacteriaceae	2.20837481	Thermoanaerobacterales Family III. Incertae Sedis	− 2.11155189					Nocardioidaceae	0.45717254
Jiangellaceae	− 2.09859541	Kordiimonadaceae	2.20535701	Jiangellaceae	− 2.09859541					Acidimicrobiaceae	0.44951922
Oscillochloridaceae	− 2.09387239	Bacillales Family X. Incertae Sedis	2.19217114	Oscillochloridaceae	− 2.09387239					Bradyrhizobiaceae	0.44735454
Thermoleophilaceae	− 2.09031284	Cyclobacteriaceae	2.12772692	Thermoleophilaceae	− 2.09031284					Nitrosomonadaceae	0.39296275
Glycomycetaceae	− 2.07081809	Aerococcaceae	2.12689459							Burkholderiaceae	0.39082058
Proteinivoraceae	− 2.06166055	Yersiniaceae	2.09222584							Chloroflexaceae	0.37964056
Fervidobacteriaceae	− 2.03464581	Corynebacteriaceae	2.08653461	Glycomycetaceae	− 2.07081809					Polyangiaceae	0.27886772
Atopobiaceae	− 2.01334894	Shewanellaceae	2.08514772	Proteinivoraceae	− 2.06166055						
Methylococcaceae	− 2.01263402	Leptotrichiaceae	2.05132180	Fervidobacteriaceae	− 2.03464581						
Rubrobacteraceae	− 1.96000736	Pseudoalteromonadaceae	2.01819354	Atopobiaceae	− 2.01334894						
Chlamydiaceae	− 1.93702817	Enterococcaceae	1.99125797	Methylococcaceae	− 2.01263402						
Acidobacteriaceae	− 1.92675589	Listeriaceae	1.97858861	Rubrobacteraceae	− 1.96000736						
Phaselicystidaceae	− 1.90446945	Neisseriaceae	1.91842247	Chlamydiaceae	− 1.93702817						
Thermotogaceae	− 1.88721594	Candidatus Midichloriaceae	1.83259270	Acidobacteriaceae	− 1.92675589						
Catenulisporaceae	− 1.86823604	Amoebophilaceae	1.82377018	Phaselicystidaceae	− 1.90446945						
Propionibacteriaceae	− 1.84788449	Staphylococcaceae	1.82131441	Thermotogaceae	− 1.88721594						
Rhodospirillaceae	− 1.84742620	Oceanospirillaceae	1.81976511	Catenulisporaceae	− 1.86823604						
Herpetosiphonaceae	− 1.84018338	Hydrogenophilaceae	1.79751167	Propionibacteriaceae	− 1.84788449						
Endomicrobiaceae	− 1.81765751	Porphyromonadaceae	1.79465903	Rhodospirillaceae	− 1.84742620						
Methylocystaceae	− 1.80270868	Idiomarinaceae	1.72635277	Herpetosiphonaceae	− 1.84018338						
Eggerthellaceae	− 1.80006110	Bartonellaceae	1.70407226	Endomicrobiaceae	− 1.81765751						
Natranaerobiaceae	− 1.78446468	Fusobacteriaceae	1.65502896	Methylocystaceae	− 1.80270868						
Polyangiaceae	− 1.74982144	Selenomonadaceae	1.61251596	Eggerthellaceae	− 1.80006110						
Acetobacteraceae	− 1.71243890	Oscillatoriaceae	1.59270684	Natranaerobiaceae	− 1.78446468						
Promicromonosporaceae	− 1.68491534	Gordoniaceae	1.49481737	Polyangiaceae	− 1.74982144						
Holophagaceae	− 1.64577497	Oscillospiraceae	1.42166431	Acetobacteraceae	− 1.71243890						
Solirubrobacteraceae	− 1.58410270	Erythrobacteraceae	1.39462321	Promicromonosporaceae	− 1.68491534						
Demequinaceae	− 1.51587393	Anaplasmataceae	1.33282779	Holophagaceae	− 1.64577497						
Desulfuromonadaceae	− 1.49409021	Piscirickettsiaceae	1.33266024	Solirubrobacteraceae	− 1.58410270						
Iamiaceae	− 1.49155615	Rickettsiaceae	1.31844354	Demequinaceae	− 1.51587393						
Solanaceae	− 1.48374210	Rhodanobacteraceae	1.30523165	Desulfuromonadaceae	− 1.49409021						
Kofleriaceae	− 1.43915616	Rhodothermaceae	1.30064279	Iamiaceae	− 1.49155615						
Lachnospiraceae	− 1.35192022	Methylophilaceae	1.26245936	Solanaceae	− 1.48374210						
Patulibacteraceae	− 1.32257929	Crocinitomicaceae	1.17630721	Kofleriaceae	− 1.43915616						
Ruminococcaceae	− 1.31007648	Oligoflexaceae	1.12332254	Lachnospiraceae	− 1.35192022						
Thermoanaerobacteraceae	− 1.29291655	Campylobacteraceae	1.10085072	Patulibacteraceae	− 1.32257929						
Rhodobacteraceae	− 1.28318200	Marinilabiliaceae	1.08196297	Ruminococcaceae	− 1.31007648						
Desulfovibrionaceae	− 1.27364265	Bifidobacteriaceae	1.06173795	Thermoanaerobacteraceae	− 1.29291655						
Thermodesulfobiaceae	− 1.26599853	Sanguibacteraceae	1.03904751	Rhodobacteraceae	− 1.28318200						
Syntrophaceae	− 1.26351223	Halomonadaceae	1.03772451	Desulfovibrionaceae	− 1.27364265						
Nocardiaceae	− 1.22659474	Rhodocyclaceae	1.03281903	Thermodesulfobiaceae	− 1.26599853						
Micropepsaceae	− 1.19447879	Clostridiaceae	1.02003134	Syntrophaceae	− 1.26351223						
Christensenellaceae	− 1.17500234	Vibrionaceae	0.92236468	Nocardiaceae	− 1.22659474						
Synergistaceae	− 1.14279030	Saprospiraceae	0.90766458	Micropepsaceae	− 1.19447879						
Mycobacteriaceae	− 1.08362391	Methylobacteriaceae	0.84578386	Christensenellaceae	− 1.17500234						
Cellulomonadaceae	− 1.07919701	Kiloniellaceae	0.84565478	Synergistaceae	− 1.14279030						
Acidimicrobiaceae	− 1.07021613	Helicobacteraceae	0.82873719	Mycobacteriaceae	− 1.08362391						
Syntrophorhabdaceae	− 1.04053432	Flammeovirgaceae	0.80633445	Cellulomonadaceae	− 1.07919701						
Heliobacteriaceae	− 1.02647674	Hydrogenothermaceae	0.69581527	Acidimicrobiaceae	− 1.07021613						
Peptococcaceae	− 0.97436149	Actinomycetaceae	0.60991335	Syntrophorhabdaceae	− 1.04053432						
Brevibacteriaceae	− 0.96188256			Heliobacteriaceae	− 1.02647674						
Sporichthyaceae	− 0.92905330			Peptococcaceae	− 0.97436149						
Tsukamurellaceae	− 0.92222812			Brevibacteriaceae	− 0.96188256						
Desulfobulbaceae	− 0.91249498			Sporichthyaceae	− 0.92905330						
Sinobacteraceae	− 0.91176299			Tsukamurellaceae	− 0.92222812						
Rhodobiaceae	− 0.80795251			Desulfobulbaceae	− 0.91249498						
Nocardioidaceae	− 0.77848134			Sinobacteraceae	− 0.91176299						
Thermodesulfobacteriaceae	− 0.75744565			Rhodobiaceae	− 0.80795251						
Intrasporangiaceae	− 0.73255817			Nocardioidaceae	− 0.77848134						
Desulfobacteraceae	− 0.69435711			Thermodesulfobacteriaceae	− 0.75744565						
Veillonellaceae	− 0.67923085			Intrasporangiaceae	− 0.73255817						
Thiotrichaceae	− 0.67213928			Desulfobacteraceae	− 0.69435711						
Beijerinckiaceae	− 0.56144362			Veillonellaceae	− 0.67923085						
				Thiotrichaceae	− 0.67213928						
				Beijerinckiaceae	− 0.56144362						

The families most abundant were Flatidae, Hymenobacteraceae and Oxalobacteraceae, and those less copious were Ktedonobacteraceae, Methanosarcinaceae and Nitrososphaeraceae.

Comparing the microbial composition of samples collected in the cultivated area during 2015 to the area sampled in 2013, PCA showed a lower variability in 2015 than the reference year at the phylum taxonomic level. *X*-axis of Fig. S6a represents the main component 1 that explains 64% of the variability. Among the 56 phyla identified, 14 appear to be significantly plentiful in 2015 and 19 scarcely represented.

At the taxonomic family level, the PCA showed a heterogeneous variability in 2015 samples (Fig. S6b). The method used identified 485 families, of which 29 were significantly copious and 139 less plentiful in 2015, with fold changes ranging between − 5.67 and 4.01 (Table 2). The families with the most differential relative abundance are Ktedonobacteraceae, Methanosarcinaceae, Nitrososphaeraceae and Williamsiaceae.

The biodiversity was evaluated at the phylum taxonomic level also comparing the cultivated area 2016 vs 2013. PCA showed a reasonable degree of variability among samples collected both in 2016 (along the principal component 2 that explains 32% of the total variability) and in 2013 (along with the main component 1 that sums up 52% of the variability) (Fig. S7a). The comparative analysis proved that 33 of the 56 identified phyla showed a significantly different abundance, as 14 were more present and 19 were in reduced quantity in the year 2016.

At the taxonomic family level, the PCA showed a high degree of diversity for the samples of 2013 and those of 2016 (Fig. S7b). The method recognised 485 families, of which 81 with significantly increased abundance (especially Stephanopyxidaceae, Coccomyxaceae, Fragilariaceae, Williamsiaceae and Chattonellaceae), and 63 with reduced quantity (in particular Methanosarcinaceae, Erysipelotrichaceae, Defluviitaleaceae and Prolixibacteraceae) in 2016 compared to 2013, with logFC between − 5.31 and 5.38 (Table 2).

The differential analysis identified the taxa associated with the sampled soils’ biological diversity, highlighting significant differences over time, especially comparing the initial situation (2013) to the first year of monitoring (2014). In 2014, we can appreciate the most significant perturbation of biodiversity. The comparative analysis showed taxa significantly different in the sampling areas, especially for the taxonomic levels of phylum and family.

The PCA results indicate a lower biological distance among the sampling points analysed in 2015 and 2016 compared to the reference year 2013, suggesting a certain degree of soil recovery.

The construction of the flowline caused changes in the bacterial community structure and their metabolic activity, with the appearance of different groups of bacteria. The analysis of 2014 sampling led to identifying bacteria commonly present in the soil where they mediate the complex metabolic processes and play a fundamental role in the functioning and stability of the system ensured by their diversification. Interesting was identifying bacterial groups naturally present in stressed soils such as bacteria belonging to Micrococcaceae, Xanthomonadaceae, Sporolactobacillaceae and Flavobacteriaceae families.

Bacterial groups resisting metals like cadmium, cobalt, zinc, chromium and mercury, or that reduce and detoxify redox-active metals, such as chromium and mercury, were in the identification list. Moreover, it was possible to identify bacterial groups belonging to the Pseudomonadaceae family, known to degrade particular harmful substances, such as naphthalene, toluene and other hydrocarbons. These beneficial bacteria are also able to degrade plastics and polystyrene to small substances.

Numerous studies have analysed soil sites contaminated with different pollutants in the past years, indicating diverse microbial populations present despite some extreme contamination conditions, showing as soils contaminated with heavy metals and hydrocarbons have undergone changes in community composition (Joynt et al., 2006; Banerjee et al., 2011). The difference in soils’ source and properties has provided different bacterial isolates, such as *Acinetobacter*, *Aeromonas*, *Aureobacterium*, *Bacillus*, *Escherichia*, *Klebsiella*, *Micrococcus*, *Pseudomonas*, *Rhodococcus* and *Stenotrophomonas* (Anderson & Cook, 2004; Jackson et al., 2005; Aksornchu et al., 2008; Vinas et al., 2005). Mathè et al. (2012) revealed how different pollutants affect the activity and diversity of endogenous microbiota. Individual isolates were tested for their ability to degrade various types of hydrocarbons (aliphatic-, mono-aromatic and polycyclic aromatic hydrocarbons) or for their capability to resist heavy metals or to increase in the presence of antibiotics. Results revealed that in contaminated sites, an increased activity of hydrocarbonoclastic bacteria occurs, supported by significant CO_2_ production. Furthermore, Alisi et al. (2009) and Sprocati et al. (2012) showed the feasibility of the remediation of a soil containing heavy metals and spiked with diesel fuel, through a bioaugmentation strategy based on the use of a microbial formula tailored to selected natives, which can effectively facilitate and speed up the bioremediation of matrices co-contaminated with hydrocarbons and heavy metals.

During the monitoring analyses in 2014, we observed an impressive presence of specific bacterial groups already found dominant in soil taken directly from oil wells (Galazka et al., 2018). Some families of Alphaproteobacteria, Rhizobiaceae, Rhodobacteraceae, Acetobacteraceae and Sphingomonadaceae were present in these samples. Bacteria that use hydrocarbons as the only carbon source were in the list and belonged to Vibrionaceae, Bacillaceae, Moraxellaceae, Mycobacteriaceae, Sphingomonadaceae, Nocardiaceae and Flavobacteriaceae families (Zhong et al., 2011). Moreover, Bacillaceae, Rhizobiaceae, Nocardiaceae, Actinomycetaceae, Nocardiaceae and Streptomycetaceae bacterial groups, associated with hydrocarbon degradation (Zhong et al., 2011; Doong & Lei, 2003; Sutton et al., 2013; Naether et al., 2012), occurred.

We also observed a differential abundance for Methylobacteriaceae, which grew on methanol and other one-carbon compounds as sources of energy and carbon, and Geobacteraceae, including several species that oxidise monoaromatic hydrocarbons, such as toluene and benzene. Then, the family Methanosarcinaceae, with the methanogenic Archaea, is most adaptable to different substrates used for energy generation and found in a wide variety of anaerobic producing methane environments (water streams, marine and hypersaline sediments, wetlands, thermal habitats, oil wells, anaerobic waste treatment systems and gastrointestinal tracts of animals) (Wegner & Liesack, 2016; Msaddak et al., 2017; Sitte et al., 2010). The presence and rapid growth of particular bacterial groups in the analysed sites indicate a natural bioremediation process to restore optimal conditions of soils altered by the flowline construction and oil pipeline presence in the underground.

## Conclusions

In conclusion, this exciting research activity needs a more in-depth investigation to understand soil bacterial populations’ activities and dynamics and the relationships between functionality and microbial diversity that underlie significant recovery and restoration processes. Although many species are necessary for the maintenance of stable processes in ever-changing ecosystems, a minimum number of species exerting a specific ecological role are essential. The greater the degree of intra- or inter-specific functional biodiversity of an ecosystem, the greater is its tolerance to perturbations and its resilience, since there will be more chances that genotypes or species can replace the functions of those disappeared.

On September 2015, a cross-sector guide for implementing the mitigation hierarchy was ready for dissemination by the Biodiversity Consultancy on behalf of International Petroleum Industry Environmental Conservation Association (IPIECA), the International Council Mining and Metals (ICMM) and the Equator Principles Association. The Cross-Sector Biodiversity Initiative (CSBI) makes available to people the Mitigation Hierarchy Guide and an executive summary, http://www.csbi.org.uk/our-work/mitigation-hierarchy-guide/ (visited on 30 December 2020).

The mission of CSBI is “developing and sharing good practices related to biodiversity and ecosystem services in the extractive industries”.

The mitigation hierarchy is an instrument designed to help users reduce, as much as possible, the negative impacts of development projects on biodiversity and ecosystem services (BES).

It includes four crucial actions — “avoid”, “minimise”, “restore” and “compensate” — and provides a best practice approach to assist in the sustainable management of natural and living resources by establishing a mechanism to balance the conservation needs with priority development. So, the monitoring work reported in this article can be considered one of the actions necessary to verify the restoration of natural equilibrium in an area that has suffered an anthropic insult.

## Supplementary Information

Below is the link to the electronic supplementary material.

Supplementary file1 (DOCX 2.05 MB)

## Data Availability

Not applicable.
