# Supplementary material for: Metagenomic monitoring of soil bacterial community after the construction of a crude oil flowline
Source: Environ Monit Assess. 2022 Jan 3;194(2):48. doi: 10.1007/s10661-021-09637-3 (PMC8724107; doi:10.1007/s10661-021-09637-3)
Supplement: Supplementary file 1 — Supplementary file1 (DOCX 2.05 MB) [file 10661_2021_9637_MOESM1_ESM.doc]

*Environmental Monitoring and Assessment*

Metagenomic monitoring of soil bacterial community after the construction of a crude oil flowline

Maria Grazia Bonomo ^1^*, Luana Calabrone^1^, Laura Scrano^2^, Sabino Aurelio Bufo^1,4^, Katia Di Tomaso^1^, Euro Buongarzone^3^ and Giovanni Salzano^1^

^1^  University of Basilicata, Department of Sciences, Potenza, Italy

^2^  University of Basilicata, Department of European Cultures, Matera, Italy

^3^  SAIPEM Spa, Fano, Italy

^4^  Department of Geography, Environmental Management & Energy Studies, University of Johannesburg, South Africa

***SUPPLEMENTARY MATERIALS***

***Soil chemical-physical analyses***

Soil samples were air dried in laminar air hood, sieved through 2 mm mesh, and used for chemical-physical analysis following standard methods.

Particle size measurements were performed by hydrometer simplified method (Gee and Bauder, 1986) and the pH was obtained by a potentiometric measurement in water suspension (Thomas, 1996). The field capacity was obtained by addition of distilled water and gravimetric measurements (Cassel and Nielsen, 1986).Water content was measured by the thermal method (Gardner, 1986) and organic matter was determined using the Walkley–Black method (Nelson and Sommers, 1996).

Electrical conductivity was measured by a conductimeter (Rhoades, 1996) to estimate the salinity.

Total carbonate content was obtained through a volumetric method (Loeppert and Suarez, 1996), phosphorus content was determined by a modified Olsen method (Kuo, 1996) and total-S (Tabatai, 1996)and total-N (Bremner, 1996)were determined by pyrolysis.

Exchangeable Ca and K were determined by Chapman (1965) and Pratt (1965) method, respectively, and cation exchange capacity analysis was performed by using Barium exchange method (Sumner and Miller, 1996).

Soil FDA, as a measure of total microbial activity, was obtained as described by Tabatai (1994) and simplified by Perucci et al. (2000) and microbial biomass was determined by fumigation method (Horwath and Paul, 1994). The mineralization coefficient was determined as described by Zibilske (1994) and Perucci et al. (2000). The soil respiration was determined using the Dumontet and Mathur method (1989) and the respiration coefficient was calculated as suggested by Anderson and Domsch (1990). Moreover, soil quality was assessed by determination of hydrocarbon content in the range C_10_ to C_40_ by gas chromatography; sum of compounds extractable with acetone/n-heptane (2+1 and sonication) that do not adsorb on a Florisil column and can be chromatographed on a non-polar capillary column with retention times between those of n-decane (C_10_H_22_) and n-tetracontane (C_40_H_82_) (ISO 16703:2004).

***References***

- Anderson, J.M.; Domsch, K.H. Application of eco-physiological quotients (qCO_2_ and qD) on microbial biomass from soils of different cropping histories*. Soil Biol Biochem* **1990**, *22*, 251-255.

- Bremner, J.M. Nitrogen-Total. In: D.L. Sparks, A.L. Page, P.A. Helmke, R.H. Loeppert, editors, Methods of Soil Analysis Part 3—Chemical Methods, SSSA Book Ser. 5.3. SSSA, ASA, Madison, WI. **1996**, p. 1085-1121.

- Cassel, D.K.; Nielsen, D.R. Field Capacity and Available Water Capacity. In: A. Klute, editor, Methods of Soil Analysis: Part 1—Physical and Mineralogical Methods, SSSA Book Ser. 5.1. SSSA, ASA, Madison, WI. **1986**, p. 901-926.

- Chapman, H.D. Total Exchangeable Bases 1. In: A.G. Norman, editor, Methods of Soil Analysis. Part 2.Chemical and Microbiological Properties, Agron.Monogr. 9.2. ASA, SSSA, Madison, WI. **1965**, p. 902-904.

- Dumontet, S.; Mathur, S.P. Evaluation of respiration based methods for measuring microbial biomass in metal-contaminated acidic mineral and organic soils*. Soil Biol Biochem* **1989**, *21,* 431-436

- Gardner, W. H. Water Content. In: A. Klute, editor, Methods of Soil Analysis: Part 1—Physical and Mineralogical Methods, SSSA Book Ser. 5.1. SSSA, ASA, Madison, WI. **1986**, p. 493-544.

- Gee, G.W.; Bauder, J.W. Particle-size Analysis1. In: A. Klute, editor, Methods of Soil Analysis: Part 1—Physical and Mineralogical Methods, SSSA Book Ser. 5.1. SSSA, ASA, Madison, WI. **1986**, p. 383-411.

- Horwath, W.R.; Paul E.A. Microbial Biomass. In: P.S. Bottomley, J.S. Angle, R.W. Weaver, editors, Methods of Soil Analysis: Part 2—Microbiological and Biochemical Properties, SSSA Book Ser. 5.2. SSSA, Madison, WI. **1994**, p. 753-773.

- Kuo, S. Phosphorus. In: D.L. Sparks, A.L. Page, P.A. Helmke, R.H. Loeppert, editors, Methods of Soil Analysis Part 3—Chemical Methods, SSSA Book Ser. 5.3. SSSA, ASA, Madison, WI. **1996**, p. 869-919.

- Loeppert, R.H.; Suarez, D.L. Carbonate and Gypsum. In: D.L. Sparks, A.L. Page, P.A. Helmke, R.H. Loeppert, editors, Methods of Soil Analysis Part 3—Chemical Methods, SSSA Book Ser. 5.3. SSSA, ASA, Madison, WI. **1996**, p. 437-474.

- Nelson, D.W., Sommers, L.E. Total Carbon, Organic Carbon, and Organic Matter. In: D.L. Sparks, A.L. Page, P.A. Helmke, R.H. Loeppert, editors, Methods of Soil Analysis Part 3—Chemical Methods, SSSA Book Ser. 5.3. SSSA, ASA, Madison, WI. **1996,** p. 961-1010.

- Perucci, P.;, Dumontet, S.; Bufo, S.A.; Mazzatura, A.; Casucci, C. Effects of organic amendment and herbicide treatment on soil microbial biomass. *Biol Fertil Soils* **2000,** *32*,17–23.

- Pratt, P.F. Potassium. In: A.G. Norman, editor, Methods of Soil Analysis. Part 2.Chemical and Microbiological Properties, Agron.Monogr. 9.2. ASA, SSSA, Madison, WI. **1965**, p. 1022-1030.

- Rhoades, J.D. Salinity: Electrical Conductivity and Total Dissolved Solids. In: D.L. Sparks, A.L. Page, P.A. Helmke, R.H. Loeppert, editors, Methods of Soil Analysis Part 3—Chemical Methods, SSSA Book Ser. 5.3. SSSA, ASA, Madison, WI. **1996**, p. 417-435.

- Sumner, M.E., Miller, W.P. Cation Exchange Capacity and Exchange Coefficients. In: D.L. Sparks, A.L. Page, P.A. Helmke, R.H. Loeppert, editors, Methods of Soil Analysis Part 3—Chemical Methods, SSSA Book Ser. 5.3. SSSA, ASA, Madison, WI. **1996**, p. 1201-1229.

- Tabatabai, M.A. Soil Enzymes. In: P.S. Bottomley, J.S. Angle, R.W. Weaver, editors, Methods of Soil Analysis: Part 2—Microbiological and Biochemical Properties, SSSA Book Ser. 5.2. SSSA, Madison, WI. **1994**, p. 775-833.

- Tabatabai, M.A. Sulfur. In: D.L. Sparks, A.L. Page, P.A. Helmke, R.H. Loeppert, editors, Methods of Soil Analysis Part 3—Chemical Methods, SSSA Book Ser. 5.3. SSSA, ASA, Madison, WI. **1996**, p. 921-960.

- Thomas, G.W. Soil pH and Soil Acidity. In: D.L. Sparks, A.L. Page, P.A. Helmke, R.H. Loeppert, editors, Methods of Soil Analysis Part 3—Chemical Methods, SSSA Book Ser. 5.3. SSSA, ASA, Madison, WI. **1996**, p. 475-490.

- Zibilske, L.M. Carbon Mineralization1. In: P.S. Bottomley, J.S. Angle, R.W. Weaver, editors, Methods of Soil Analysis: Part 2—Microbiological and Biochemical Properties, SSSA Book Ser. 5.2. SSSA, Madison, WI. **1994**, p. 835-863.

**Table S1**. Chemical-physical determinations of forest area samples.


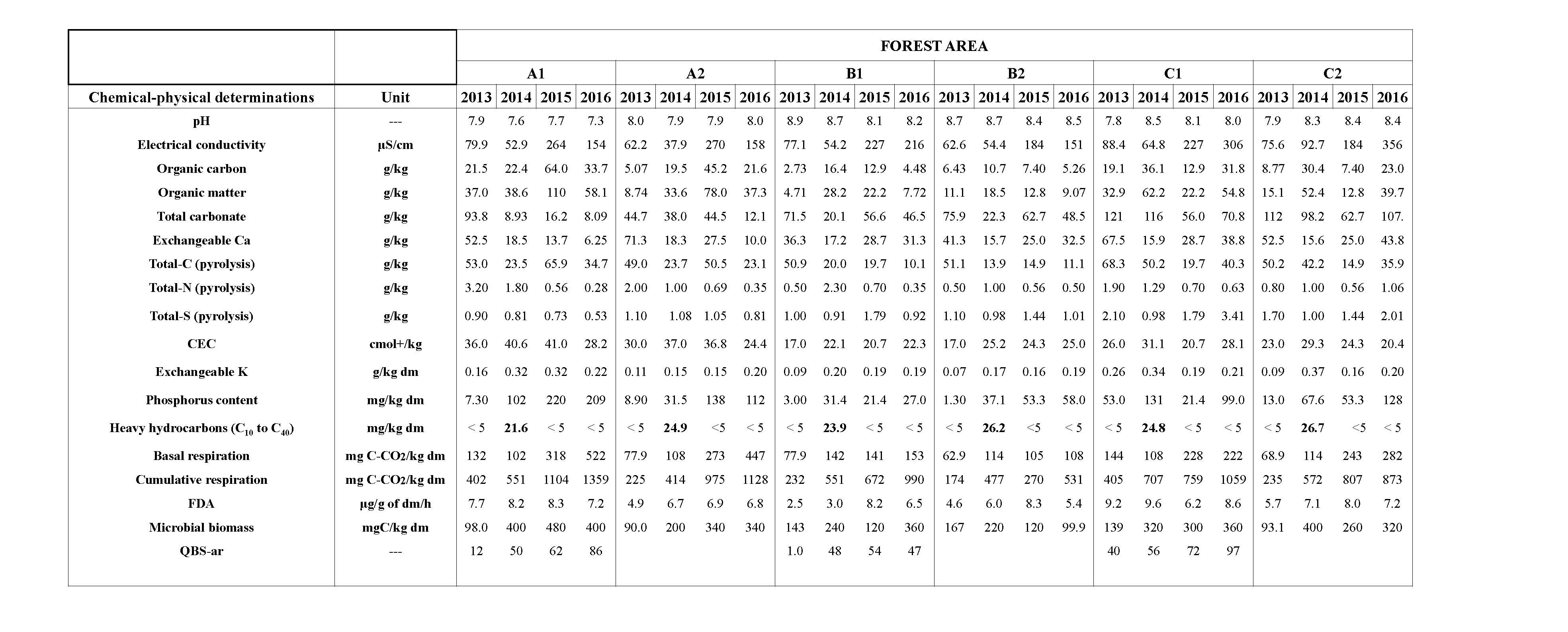


**Table S2**. Chemical-physical determinations of cultivated area samples.


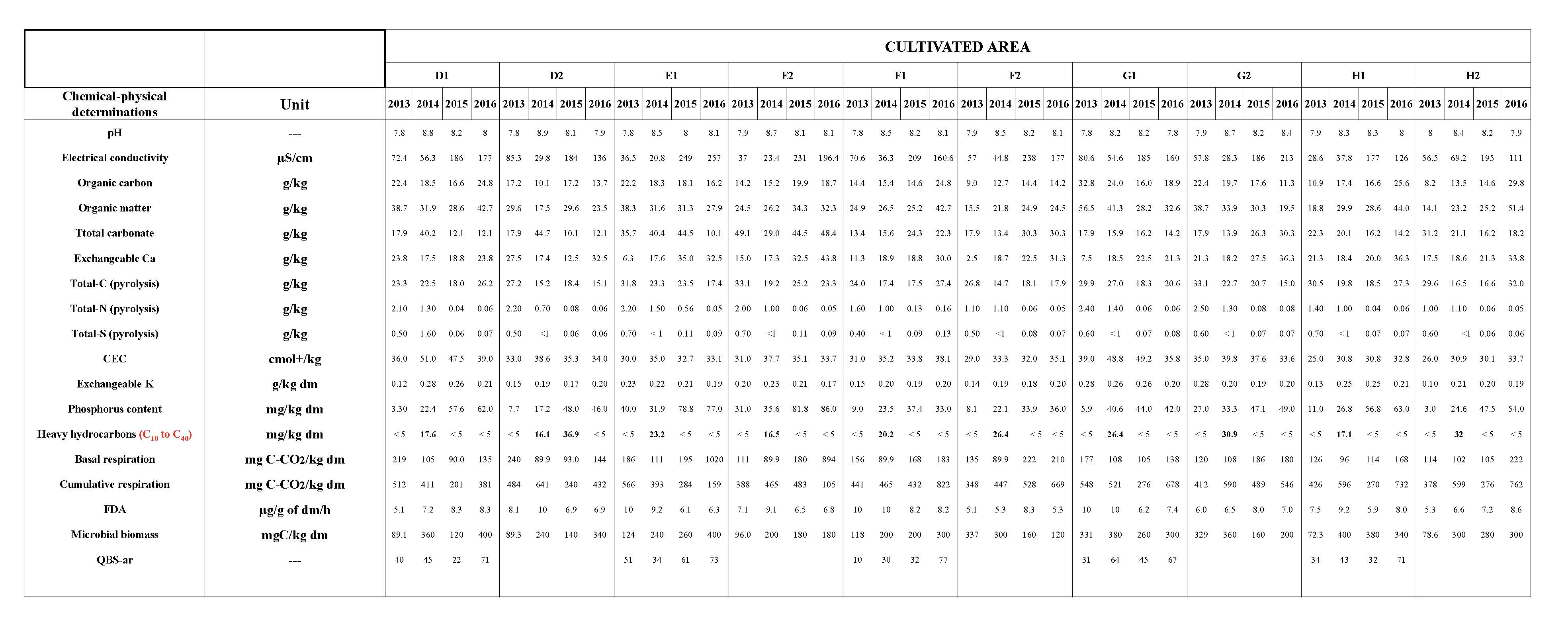


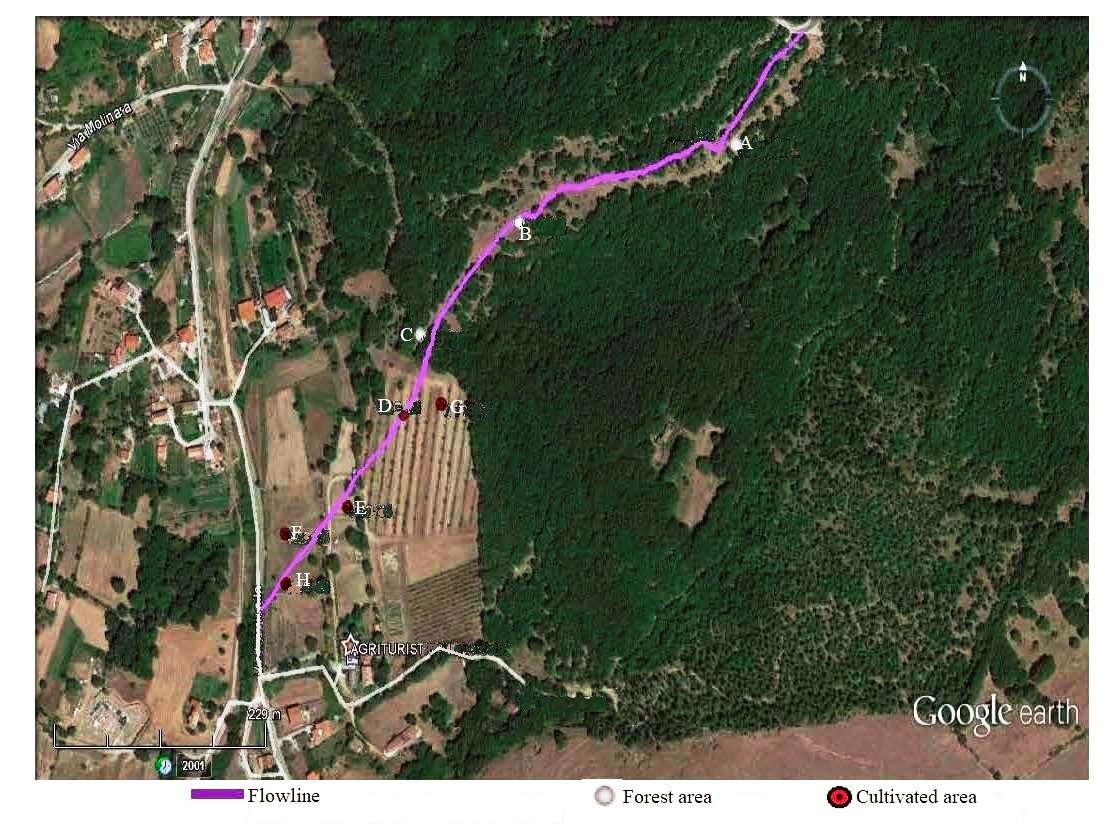


**Figure S1**. Google earth picture of the sampling area


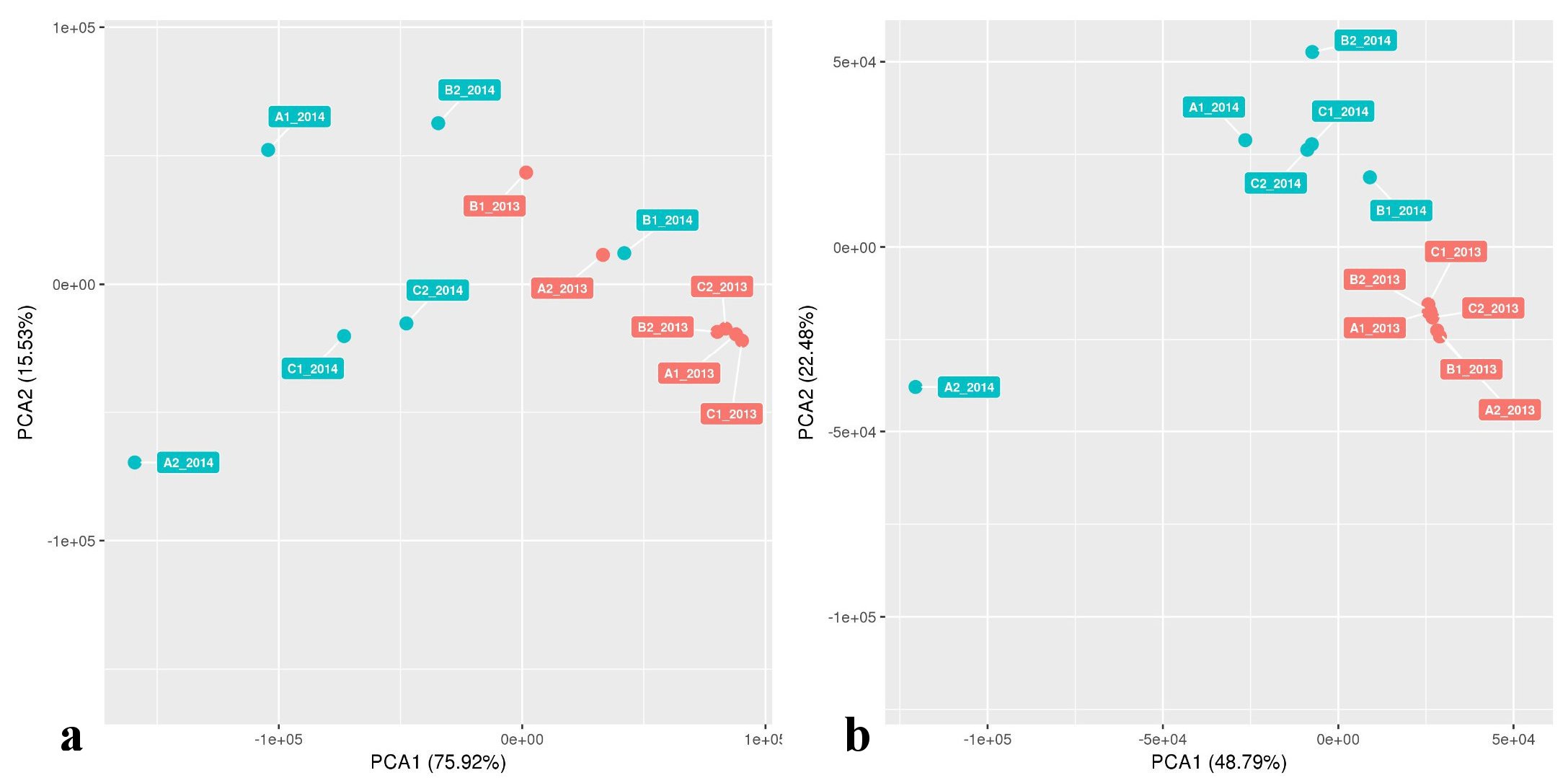


**Figure S2.** Comparison between the sampling of the forest area in 2014 and the sampling of 2013: PCA distribution according to the distance among samples at the phylum (**a**) and family (**b**) taxonomic level.


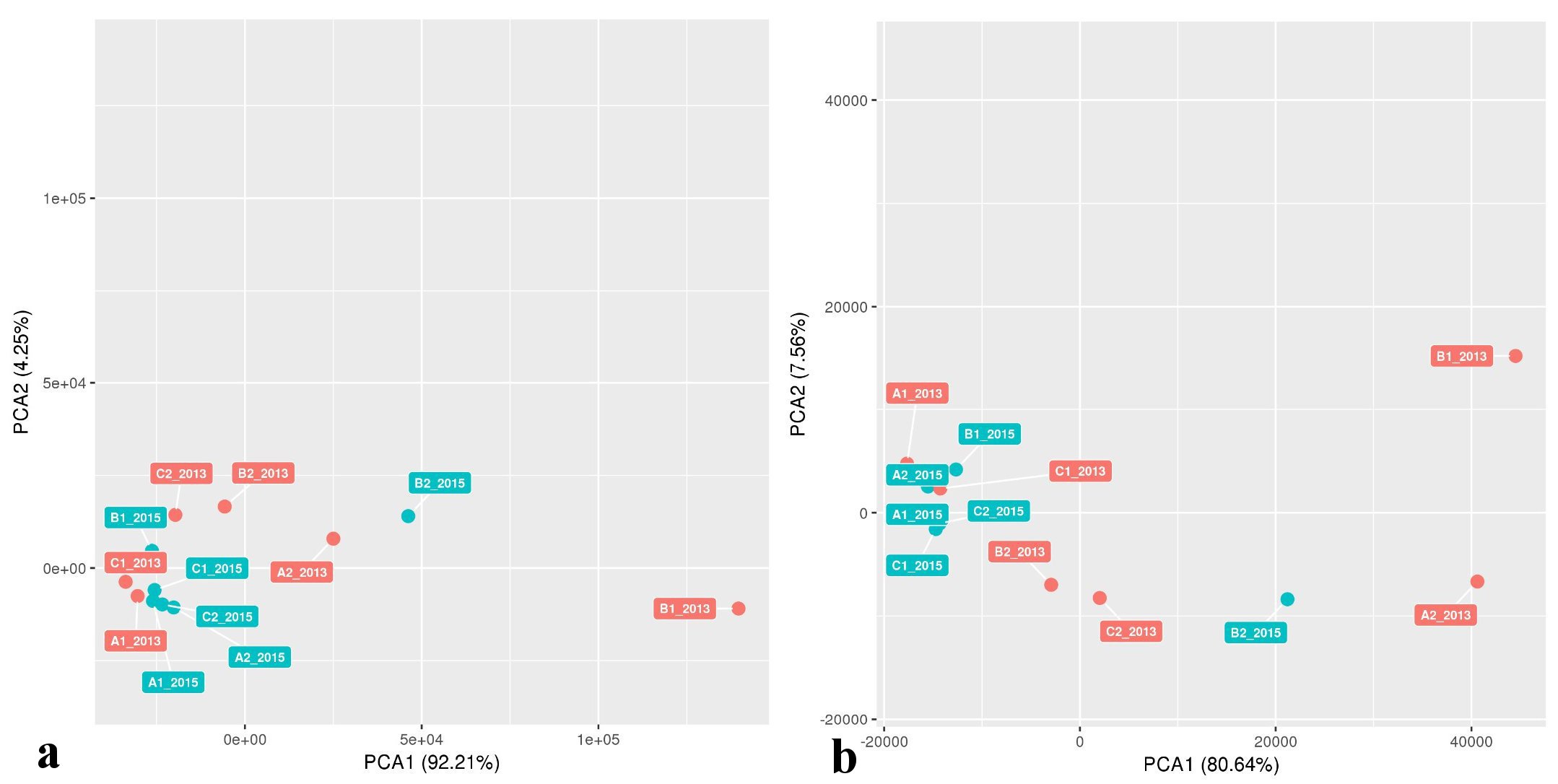


**Figure S3.** Comparison between the sampling of the forest area in 2015 and the sampling of 2013: PCA distribution according to the distance among samples at the phylum (**a**) and family (**b**) taxonomic level.


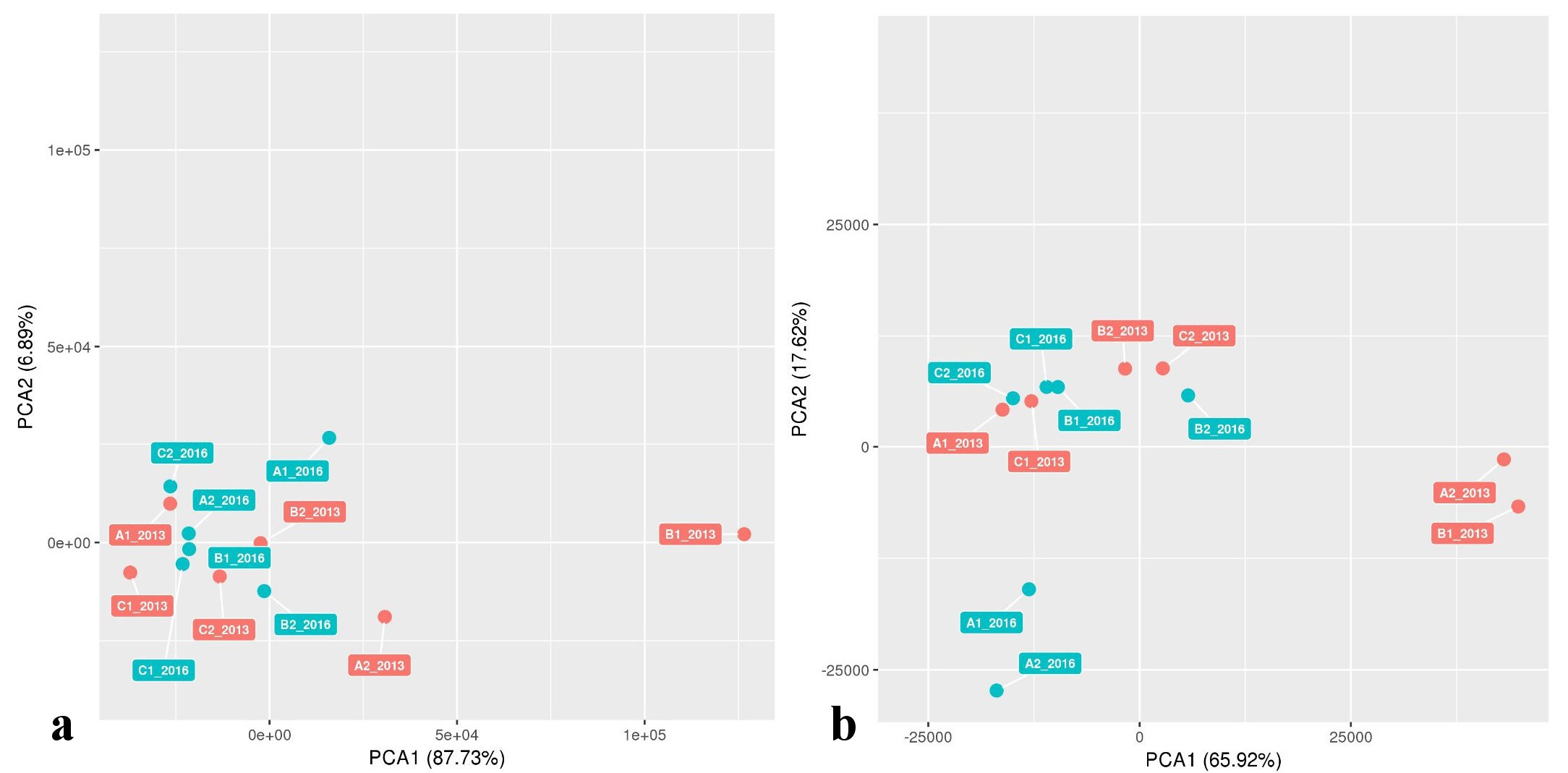


**Figure S4.** Comparison between the sampling of the forest area in 2016 and the sampling of 2013: PCA distribution according to the distance among samples at the phylum (**a**) and family (**b**) taxonomic level.


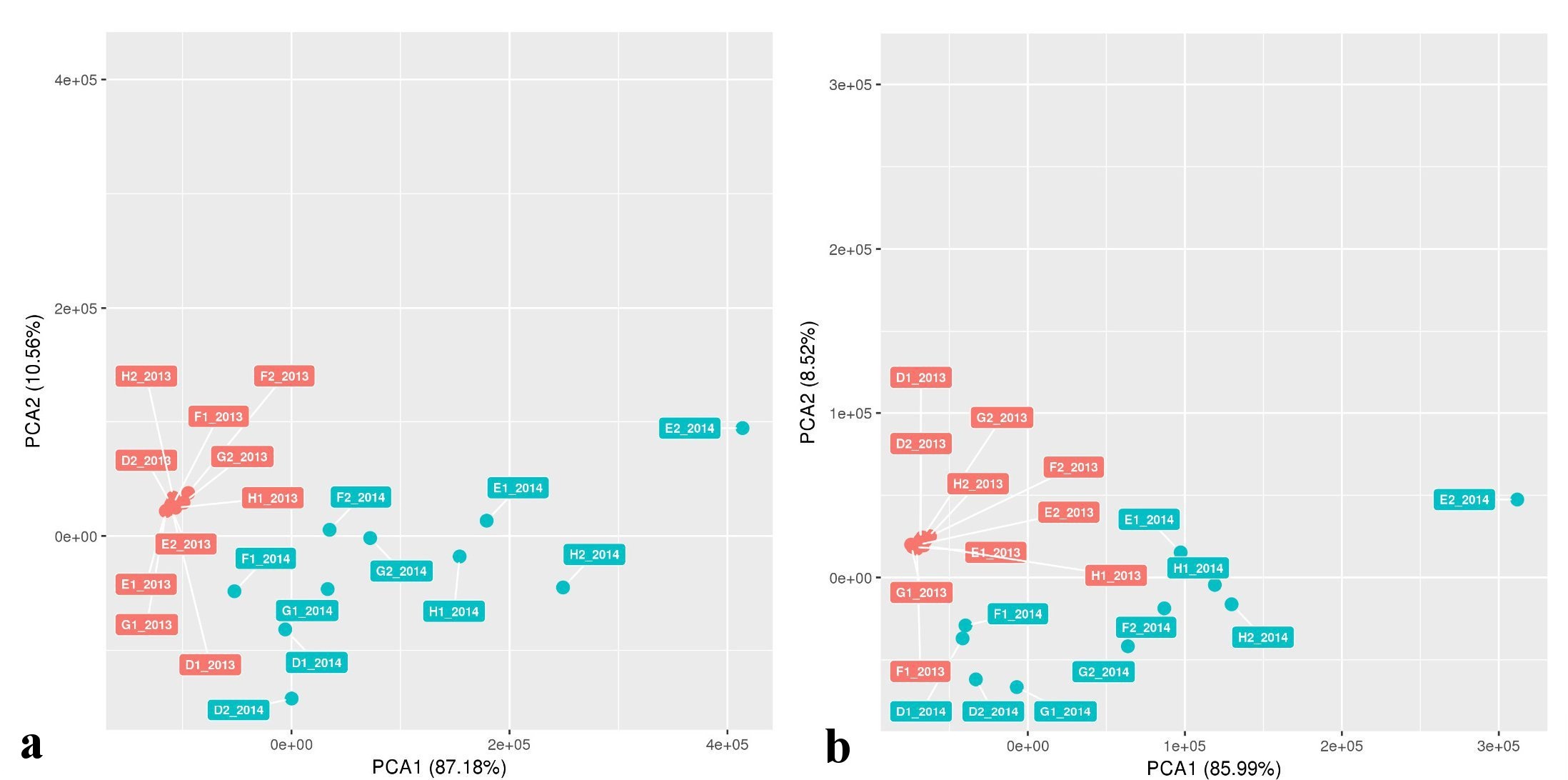


**Figure S5.** Comparison between the sampling of the cultivated area in 2014 and 2013: PCA distribution according to the distance among samples at the phylum (**a**) and family (**b**) taxonomic level.


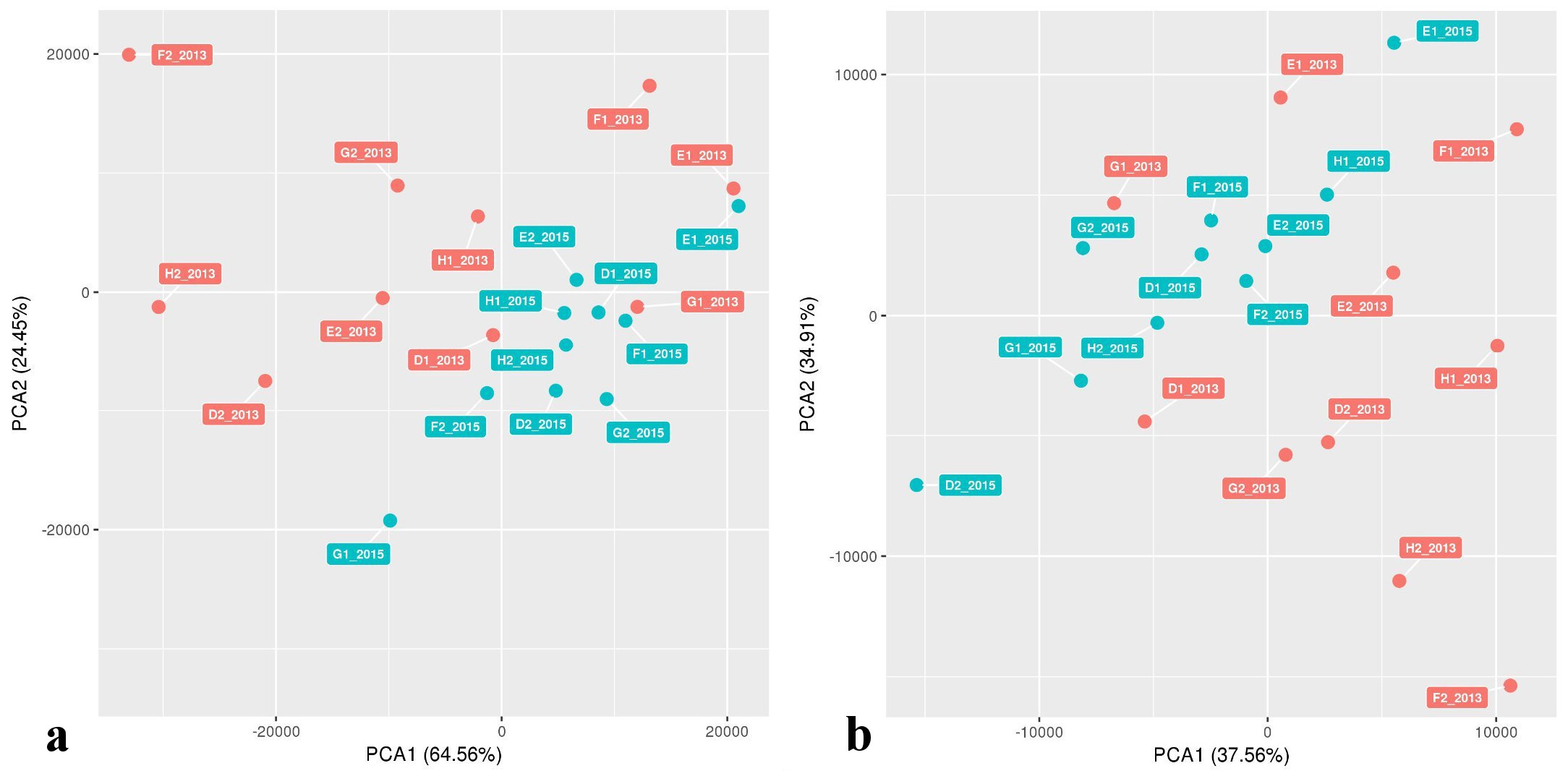


**Figure S6.** Comparison between the sampling of the cultivated area in 2015 and in 2013: PCA distribution according to the distance among samples at the phylum (**a**) and family (**b**) taxonomic level.


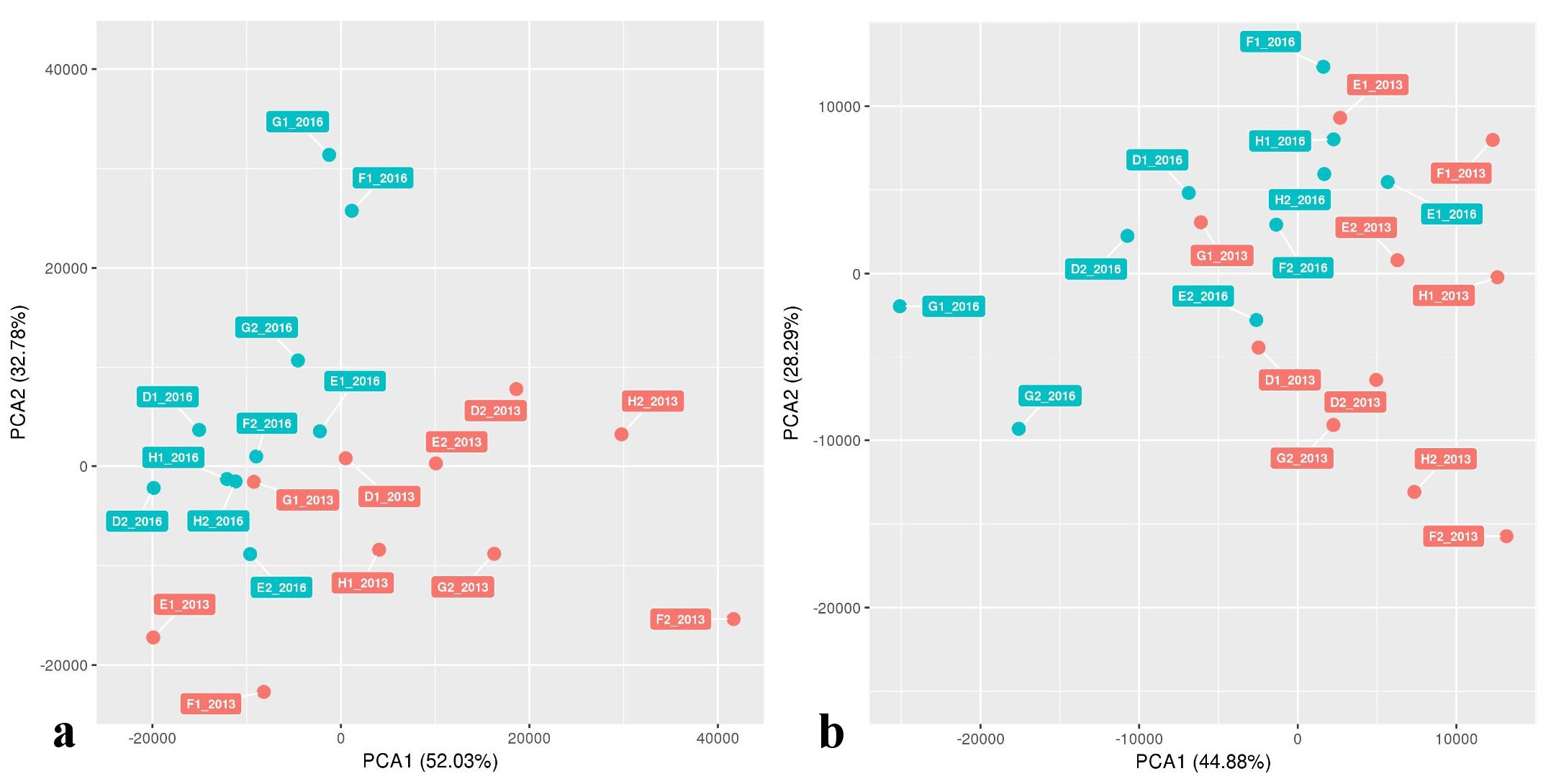


**Figure S7.** Comparison between the sampling of the cultivated area in 2016 and 2013: PCA distribution according to the distance among samples at the phylum (**a**) and family (**b**) taxonomic level.
